# PM_2.5_ induce lifespan reduction, insulin/IGF-1 signaling pathway disruption and lipid metabolism disorder in *Caenorhabditis elegans*

**DOI:** 10.3389/fpubh.2023.1055175

**Published:** 2023-02-02

**Authors:** Wenjing Zhang, Zinan Li, Guojun Li, Ling Kong, Haiming Jing, Nan Zhang, Junyu Ning, Shan Gao, Yong Zhang, Xinyu Wang, Jing Tao

**Affiliations:** ^1^Beijing Center for Disease Prevention and Control, Beijing, China; ^2^School of Public Health, Capital Medical University, Beijing, China

**Keywords:** PM_2.5_, *Caenorhabditis elegans*, lifespan, healthspan, RNA-Sequencing, gene homology

## Abstract

**Introduction:**

Exposure to fine particulate matter (PM), especially PM_2.5_, can induce various adverse health effects in populations, including diseases and premature death, but the mechanism of its toxicity is largely unknown.

**Methods:**

Water-soluble components of PM_2.5_ (WS-PM_2.5_) were collected in the north of China in winter, and combined in two groups with the final concentrations of 94 μg/mL (C_L_ group, AQI ≤ 100) and 119 μg/mL (C_H_ group, 100 < AQI ≤ 200), respectively. The acute and long-term toxic effects of WS-PM_2.5_ samples were evaluated in several aspects such as development, lifespan, healthspan (locomotion behavior, heat stress tolerance, lipofucin). DAF mutants and genes were applied to verify the action of IIS pathway in WS-PM_2.5_ induced-effects. RNA-Sequencing was performed to elucidate the molecular mechanisms, as well as ROS production and Oil red O staining were also served as means of mechanism exploration.

**Results:**

Body length and lifespan were shortened by exposure to WS-PM_2.5_. Healthspan of nematodes revealed adverse effects evaluated by head thrash, body bend, pharyngeal pump, as well as intestinal lipofuscin accumulation and survival time under heat stress. The abbreviated lifespan of *daf-2(e1370)* strain and reduced expression level of *daf-16* and *hsp-16.2* indicated that IIS pathway might be involved in the mechanism. Thirty-five abnormally expressed genes screened out by RNA-Sequencing techniques, were functionally enriched in lipid/lipid metabolism and transport, and may contribute substantially to the regulation of PM_2.5_ induced adverse effects in nematodes.

**Conclusion:**

WS-PM_2.5_ exposure induce varying degrees of toxic effects, such as body development, shorten lifespan and healthspan. The IIS pathway and lipid metabolism/transport were disturbed by WS-PM_2.5_ during WS-PM_2.5_ exposure, suggesting their regulatory role in lifespan determination.

## Introduction

Suffering from air pollution is one of the major common public health issues in recent decades. Air pollution has been deemed as a public health problem since about 90% of the population in the world live in areas where air quality exceeds the limits of WHO guidelines (1). According to Global Burden of Disease Research Report (2), persistent and intense air pollution has become an immense burden on healthcare and health economy globally. By 2019, air pollution has been the fourth largest risk factor in the world (3). Particulate matter (PM), especially those with a diameter of 2.5 microns or less (also called fine particulate matters or PM_2.5_), is a common proxy indicator for air pollution, which affects more people than any other pollutants. Epidemiological studies and animal studies have shown that there is a close, quantitative relationship between PM_2.5_ exposure and increased morbidity, mortality and the incidence of various diseases, e.g., respiratory diseases, cardiovascular diseases, immune system diseases and metabolic diseases, cognitive impairment (4–7). Moreover, ambient PM_2.5_ exposure has been identified as an important risk factor for premature death or chronic stress which leads to a reduction of Life Expectancy (LE) or Disability-Adjusted Life Years (DALY) in populations (8–13), whereas the regulatory mechanism underlying remains obscure. In addition, Health-Adjusted Life Expectancy (HALE) is a comprehensive evaluation index for population health that takes into account death and disability, which was recommended by WHO as a comprehensive measurement index for evaluating population health (14).

The current literature reported that ambient fine particulate matters significantly reduces the HALE of human population, but the impact and its regulatory mechanism on healthspan in model organisms and human HALE are all unknown, which deserves further investigation.

Nematode *Caenorhabditis elegans* (*C. elegans*) has been recognized as a powerful model organism for studying lifespan, for elucidating conserved pathways and processes relevant to human aging. In the past decays, *C. elegans* has been employed as experimental model in toxicity evaluation of PM_2.5_, suggesting that heavy metal and polycyclic aromatic hydrocarbons (PAHs) components in PM_2.5_ exposure induces deficits in development, reproduction, locomotion behavior, defecation behavior, lifespan, intestinal homeostasis or reactive oxygen species (ROS) elimination, in parent or progeny of nematodes. These abnormal manifestations may be related to oxidative stress, intestinal damage, dysregulation of microRNA expression and disruption of specific signaling pathways (15–19).

The health effect of PM_2.5_ samples varies among regions where they were collected. There is a correlation between the occurrence of premature death and air pollutants from seven sources in urban and rural environments (natural, industrial, land transportation, residential and commercial energy use, power generation, biomass combustion and agricultural sources). One of the main reasons underlying this phenomenon was thought to be the diverse and complicated chemical composition of those region-specific PM_2.5_ samples. In addition, different chemical components of PM_2.5_ show different correlations with specific health effects, long-term exposure to sulfates, ammonium and sodium ions is thought to be the major cause of reduction in life expectancy (5, 6).

Previously, we have collected and analyzed the components of four kinds of pollution sources (traffic, coal, industry and dust) of fine particulate matter from one of the cities in North China, and pilot study found that fine particulate matter has adverse effects on lifespan and healthspan of *C. elegans*. This study designed to firstly detect the toxicity effect phenotype of fine particulate matter to healthspan of *C. elegans*, and discriminate the healthspan toxicity effect-oriented key toxic components and the dominate pollution source, by using the acknowledged *C. elegans* healthspan assay. Secondly, the fine particulate matter-induced differentially expressed genes/proteins relevant to the healthspan impact phenotype will be identified and verified by the RNA-Seq and quantitative differential proteomics techniques, followed by the analysis of Gene Ontology (GO) and Pathway analysis, to explore the underlying molecular regulatory mechanism of ambient fine particulate matter on *C. elegans* healthspan. Moreover, the differential expressed genes/proteins in *C. elegans* will be compared the homology with those published human age-associated gene profile collected in the database of GenAge (http://genomics.senescence.info/genes), further narrowing down the candidate gene profiles related to human aging induced by ambient fine particulate matter. The results will provide the clue and critical evidence for future comprehensive popular health risk assessment of fine particulate matter and intervention strategies.

## Materials and methods

### *C. elegans* strain and maintenance

*C. elegans* strains wild-type N2, CB1370: *daf-2(e1370)*, CF1038:*daf-16(mu86)* were purchased from *Caenorhabditis* Genetics Center (CGC). Nematodes were maintained on Nematode Growth Medium (NGM) agar plates carrying a lawn of *Escherichia. coli* OP50. Synchronized nematode were achieved by bleaching treatment of gravid hermaphrodites and eggs were allowed to hatch overnight in S-basal buffer (20).

### PM_2.5_ samples

WS-PM_2.5_ samples were collected in a region of north China in winter. The PM_2.5_ sampling membranes was placed in a 15 mL centrifuge tube, adding 14 mL ultrapure water (resistivity 18.2 MΩ · cm), and performing ultrasonic extraction for 2 h (ice cubes were added to the ultrasound system to prevent the water temperature from rising). After vortexing for 5 min, the extract was filtered through a 0.1 μm filter membrane before analysis. Every sampling membrane was processed twice, and the ultrasonically dissolved solutions are mixed to detection of F^−^, Cl^−^, ${\text{NO}}_{2}^{-}$, ${\text{NO}}_{3}^{-}$, ${\text{SO}}_{4}^{2-}$, Br^−^, H_2_${\text{PO}}_{4}^{-}$, ${\text{ClO}}_{4}^{-}$ and ${\text{NH}}_{4}^{+}$. The processed solution was stored at 4°C. Three groups were set up according to different exposure doses including blank control group (ddH_2_O), low concentration of PM_2.5_ aqueous solution (low concentration group, C_L_ group, AQI ≤ 100) and high concentration group (high concentration group, C_H_ group, 100 < AQI ≤ 200), whereas the samples were combined in each group for the further study of short-term and long-term toxic effects. In the experimental system, the final concentrations of WS-PM_2.5_ in both groups were 94 μg/mL(C_L_ group) and 119 μg/mL(C_H_ group), respectively.

### Lethality and development

The synchronized N2 were incubated on NGM seeded with OP50 to L4 larvae and approximately 40 worms/well were transferred to a 96-well plate. Nematodes were scored as dead or survival if they responded to a stimulus by a platinum wire needle under a microscope, marked as N_0_ and N_24_. Mortality lethal rate = (N_24_ – N_0_)/N × 100%. The assay was repeated three times.

In order to evaluate development parameters following WS-PM_2.5_ exposure, 20 nematodes in each group were selected randomly and observed under a fully inverted fluorescent phase-contrast microscope, amplified 10 × 10 times and determined the body length and width of each nematode using ZEN software. The assay was repeated three times.

### Lifespan

About 60 synchronized L4 nematodes were transferred into the 3 parallels 96-well plate and incubated at 20°C. Half of the liquid culture system was replaced per 24 h to prevent the generation of impurities and interference with the observation of the nematode's life state. The survival of nematode was recorded daily until the nematodes were totally dead.

### Locomotion behavior

The locomotion behavior was assessed by the endpoints of head thrash, body bend and pharyngeal pump rates. The methods were performed as described previously (21). A head thrash was defined as a change in the direction of bending at the mid body. A body bend was counted as a change in the direction of the part of nematodes corresponding to posterior bulb of the pharynx along the y axis, assuming that nematode was traveling along the x axis (the initial direction of posterior bulb of pharynx). Fifty nematodes were examined per treatment, and three replicates were performed.

The pharyngeal pumping rate reflected the food intake ability of *C. elegans*. The nematode was amplified under an automatic inverted fluorescence phase contrast microscope to clearly observe the movement of the pharyngeal pump. Each nematode was recorded for 30 s by ZEN2012 software, and then the video was slowed down at 8 fps to count the movement rate of the nematode pharyngeal pump. Twenty or more nematodes were counted for each dose group.

### Heat stress resistance assay

Resistance to lethal heat stress derived from a heat stress environment (35°C) was conducted 5-d and 10-d post-exposure. Sixty individuals from each treatment group were placed on a 35 mm blank NGM plate seeded with *E. coli* OP50, and deaths were recorded every 2 h until all the worms were dead. The standard of death is to touch the body or the head with a platinum needle without thrashing, and the standard of judgment is uniform.

### Production of lipofucin

Nematodes were treated with 94 μg/mL (C_L_ group) and 119 μg/mL (C_H_ group) WS-PM_2.5_. At 5 d and 10 d, the intestinal autofluorescence of each nematode was observed under an upright fluorescence phase contrast microscope. The images were captured by ZEN2012 software in channel DAPI and the average pixel density was calculated to determine the accumulation of lipofuscin in the nematode intestine.

### RNA extraction, DGE library preparation, and sequencing

A total amount of 3 μg RNA per sample which passed through the quality test was used as input material for the RNA sample preparations. Sequencing libraries were generated using NEBNext Ultra RNA Library Prep Kit for Illumina (NEB, USA) following manufacturer's recommendations and index codes were added to attribute sequences to each sample. The library quality was assessed on the Aillient Bioanalyzer 2100 system. The clustering of the index-coded samples was performed on a cBot Cluster Generation System using TruSeq PE Cluster Kit v3-cBot-HS (Illumina) according to the manufacturer's instructions. After cluster generation, the library preparations were sequenced on the Illumina Hiseq 4000 platform and 50 bp single-end reads (raw reads) were generated. Clean reads were obtained by processing raw reads in Fastq format through in-house perl scripts to remove reads containing adapter and ploy-N as well as low quality reads.

During the mapping process, *C elegans* genome used as reference genome and gene model annotation files were downloaded from Genomics Institute of University of California, Santa Cruz (UCSC)'s website (http://hgdownload.soe.ucsc.edu/downloads.html#nematodes) directly. The index of the reference genome was built using Bowtie (v2.2.3) and clean reads were aligned to the reference genome for mapping by using TopHat (v2.0.12). Quantification of gene expression level was achieved by using HTSeq v0.6.1 to count the reads numbers uniquely mapped to each gene. And then fragment per kilobase of exon per million fragment (FPKM) values of each gene was calculated based on the length of the gene and reads count mapped to this gene.

Prior to differential gene expression analysis, for each sequenced library, the read counts were adjusted by edgeR program package through one scaling normalized factor. Differential expression analysis of two conditions was performed using the DEGSeq R package (1.20.0). The *P*-values were adjusted using the Benjamini and Hochberg method. Corrected *P*-value of 0.05 and log2 (Fold change) of 1 were set as the threshold for significantly differential expression.

Gene Ontology (GO) and KEGG enrichment analysis of differently expressed genes (DEGs) was implemented by the GOseq R package and KOBAS software, respectively, terms with corrected *P*-value < 0.05 were considered significantly enriched by DEGs. The protein-protein interaction (PPI) network of proteins expressed by DEGs was predicted and analysis by using STRING database (https://string-db.org/).

The metadata including clean reads in a fastq format and FPKM value of each sample could be accessed in Gene Expression Omnibus (GEO) database (Accession No.: GSE214215, https://www.ncbi.nlm.nih.gov/geo/query/acc.cgi?acc=GSE214215).

### Lifespan in *daf-2*(*e1370*) and *daf-16*(*mu86*) and RT-PCR of N2 nematode

WS-PM_2.5_ with concentration of 119 μg/mL (C_H_ group) was exposed to *daf-2(e1370)* and *daf-16(mu86)* mutants, and the method is the same as the WS-PM_2.5_ exposure method of wild type nematodes.

Real-time PCR was conducted to measure expression levels of *daf-16* and *hsp-16.2* after 5 d- and 10 d-exposure in N2.

### Measurement of intracellular reactive oxygen species

Reactive oxygen species (ROS) levels were measured with 2′, 7′-dichlorofluorescein diacetate (DCFH-DA). Nematodes were transferred to black 96-well plates containing M9 and incubated for 1 h with 20 μM DCFH-DA (final concentration) at 20°C. ROS-associated fluorescence levels were measured in a microplate reader at 485 nm excitation and 520 nm emission wavelengths at room temperature. Data were normalized to protein content determined by the Bradford method. Analyses were carried out in duplicate and the experiment was independently repeated four times.

### Lipid staining

As described (22), Oil Red O was used to evaluate the lipid staining in parenchymal cells of C.elegans after 5 days exposure to PM_2.5_. (1) The nematodes was aspirated into a 1.5 mL centrifuge tube and washed twice with PBS (phosphate buffer solution). (2) Adding 1 mL of propylene glycol, dehydrate for 20 min and discard the supernatant. (3) Adding about 1 mL of Oil Red O solution and incubate overnight with shaking. (4) Centrifuging and discarding the staining solution. Add 60–80% propylene glycol and centrifuge at 5,000 rpm for 3 min. (5) After washing twice with distilled water, the nematodes were dropped on a 2% agarose pad, and photographed under a fully automatic upright fluorescence phase contrast microscope.

### Statistical analysis

Statistical analysis was performed using SPSS 19.0 (IBM, Inc., New York, USA), and all data were expressed as the mean ± SD. One-way ANOVA with Bonferroni test as the *post-hoc* comparison was used to determine the statistical difference between means of continuous variable. Kruskal-Wallis rank sum test was employed to analyse data of survival time. When the *p*-values were < 0.05 or 0.01 or 0.001, the data were considered statistically significant, that is, ^*^p < 0.05, ^**^p < 0.01, and ^***^p < 0.001 compared with the control group.

## Results

### Lethality, development delay

Acute toxicity of WS-PM_2.5_ was investigated at 24 h after exposure. WS-PM_2.5_ at concentrations of 94 and 119 μg/mL did not induce lethal effect on nematodes, but resulted in a slight development delay. The body length and body width were reduced by 3 and 4% in C_H_ group.

### WS-PM_2.5_ shortens lifespan of N2 *C. elegans*

**Figure 1:**
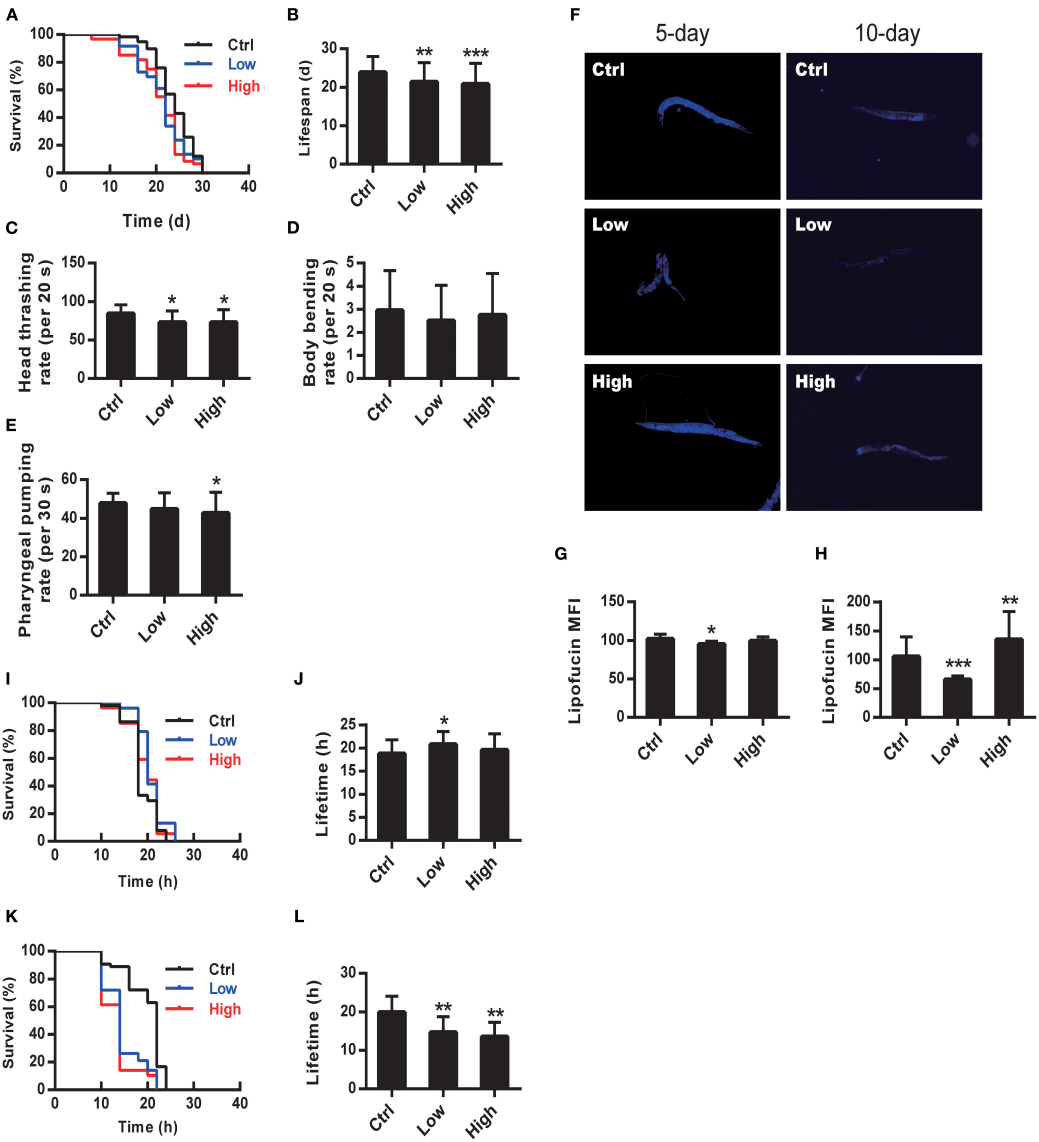
WS-PM_2.5_ exposure reduces lifespan and healthspan in *C. elegans*. **A–H** N2 nematodes were exposed to C_L_ sample (with AQI lower than 100 and concentration at 94 μg/mL) or C_H_ sample (with AQI ranging from 100 to 200 and concentration at 119 μg/mL) throughout their life circles. Survival curve **(A)** and average lifespan **(B)** were recorded and calculated. Head thrashing **(C)**, body bending **(D)** and pharyngeal pumping **(E)** rates were recorded respectively, right after the first 24-h exposure. Accumulation of lipofucin was measured by using fluorescence microscopy after the 5 d (left column of **F, G**) or 10 d (right column of **F, H**) of exposure. **I–L** The heat stress tolerance of N2 nematodes was indicated by the lifespan under heat-stress after WS-PM_2.5_ exposure. Nematodes were exposed to C_H_ or C_L_ WS-PM_2.5_ for 5 d **(I, J)** or 10 d **(K, L)**, then cultured under the heat-stress environment (35°C). Survival curve **(I, K)** and average lifetime **(J, L)** were recorded and calculated. In all groups, 60 nematodes were randomly selected for experimental manipulation. All values are given as mean ± SD. Statistical significance was marked with asterisk on the top of bars of each groups vs. corresponding controls, *p < 0.05, **p < 0.01, ***p < 0.001; F: **(C)** 4.349, **(D)** 0.354, **(E)** 2.227, **(G)** 12.470, **(H)** 26.861. “Low” and “High” represents C_L_ and C_H_ group, respectively.

Nematodes N2 were exposed to WS-PM_2.5_ at concentrations of 94 and 119 μg/mL, respectively, and ddH_2_O as negative control. Then the lifespan of each group was measured. Survival curve (Figure 1A) of both two WS-PM_2.5_ treated groups shifted to the left. Consistently, the average lifespan of two WS-PM_2.5_ treated groups decreased by 10.63% (C_L_ group) (*P* < 0.05) and 12.82% (C_H_ group) (*P* < 0.01) (Figure 1B).

### Behavior Toxicity of WS-PM_2.5_ in wild-type nematodes

Stamper et al. (23) judged survival status by observing the nematode's athletic ability, and some researchers confirmed the early nematode change in locomotion function predicts subsequent life and health life states (24, 25). We investigated the effect of WS-PM_2.5_ exposure on the induction of toxicity in nematodes. Head thrashing and body bending frequency, as well as pharynx pumping rate were utilized as the endpoints of locomotion behavior. We observed more significantly decreased head thrash in wild-type nematodes exposed to both WS-PM_2.5_-treated groups (*P* < 0.05) (Figure 1C). Body bending frequencies between WS-PM_2.5_ exposure groups and the control group were not statistically obvious (*P* > 0.05) (Figure 1D). Pharygneal pump frequency in 94 μg/mL group did not differ significantly (*P* > 0.05), while the indicator in C_H_ group statistically decreased by 11% (*P* < 0.05) (Figure 1E).

### Exposure to WS-PM_2.5_ induced a reduction in thermotolerance

In nematodes and other organisms, changes in longevity are generally proportional to their ability to resist environmental stresses, including thermal stress and oxidative stress, and changes in the ability to resist stress may be one of the explanations for changes in longevity (26, 27). In the study, after a 5-d exposure, the average survival time of nematodes in the WS-PM_2.5_-treated groups were not significantly different from that in the control group (Figures 1I, J). On the 10th day, the average survival time of nematodes in the WS-PM_2.5_-treated group was reduced by 26.29% (C_L_ group) and 31.25% (C_H_ group), respectively, under heat stress (*P* < 0.01) (Figures 1K, L), suggesting that WS-PM_2.5_ reduced the thermotolerance of *C. elegans*, which may further affect the lifespan.

### Effects of WS-PM_2.5_ exposure on the accumulation of intestinal lipofuscin

In nematodes, intestinal tract plays a key role in regulating toxicity and migration of toxins, and the effect of WS-PM_2.5_ exposure can be studied by using intestinal spontaneous fluorescence as an endpoint, which is caused by lysosomal deposits of lipofuscin, accumulating over time and reflecting the degree of oxidative damage in nematodes (28, 29). The nematodes were treated with WS-PM_2.5_ and captured by an upright fluorescence phase contrast microscope on the 5 and 10th day, respectively to compare the accumulation of lipofuscin between different groups through intestinal autofluorescence (Figure 1F). The results showed that the relative autofluorescence intensity of the nematode in C_H_ group was significantly higher than that in the control group on day 10 (*P* < 0.01) (Figure 1H), suggesting that PM_2.5_ exposure can significantly increase the accumulation of lipofuscin in the nematode.

### WS-PM_2.5_-induced gene expression alterations

#### Global gene expression analysis shows that the homeostasis of lipid transport and metabolism is disturbed by WS-PM_2.5_ exposure

**Figure 2:**
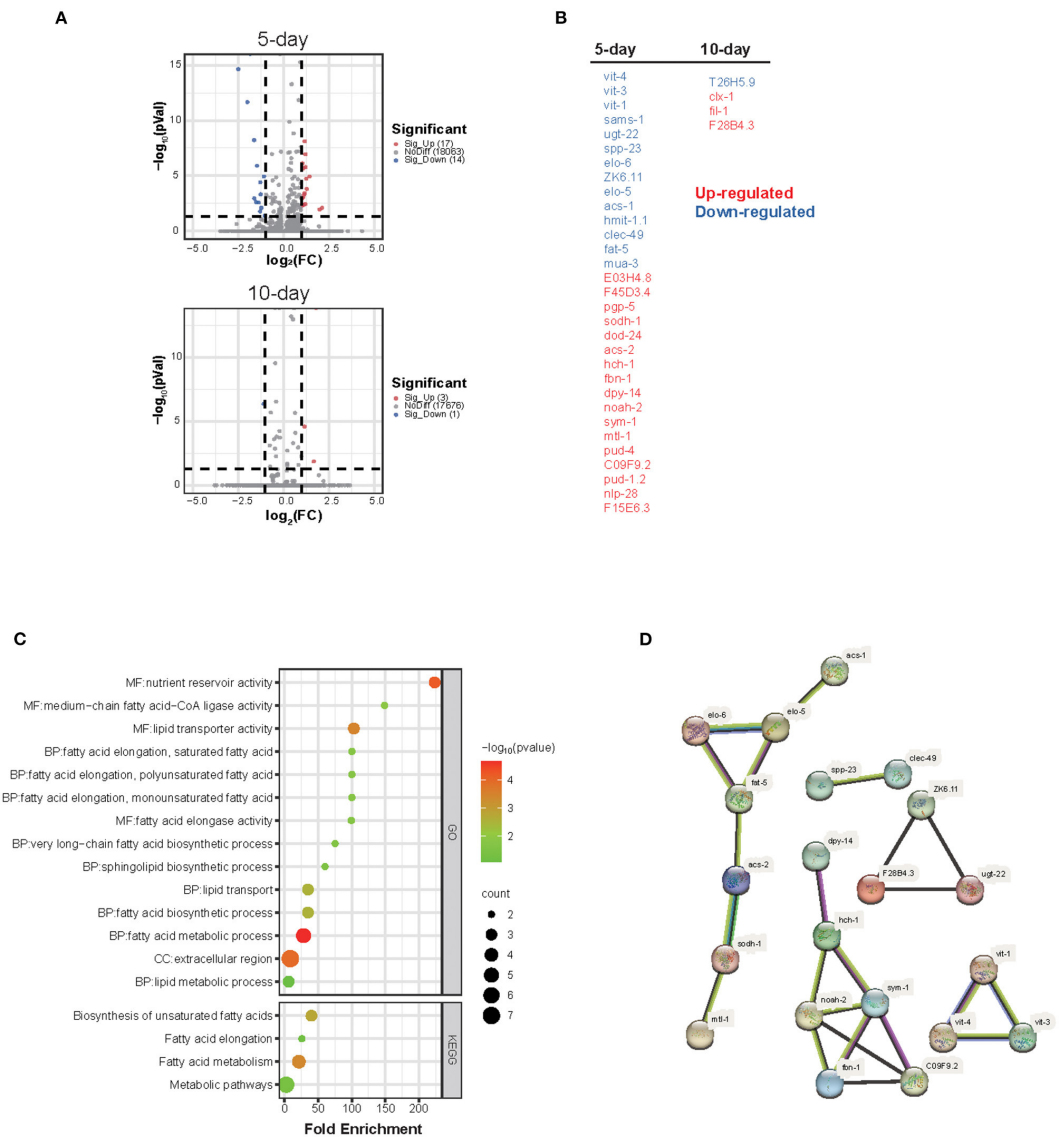
Global gene expression analysis shows that the homeostasis of lipid transport and metabolism is disturbed by WS-PM_2.5_ exposure. Global gene expression of C_H_ WS-PM_2.5_-treated nematodes as well as control nematodes were determined by RNA-Sequencing technology. **(A)** Volcano plots show the numbers of DEGs between treated and control nematodes at the 5th day **(A)** or the 10th day **(B)** during the C_H_ WS-PM_2.5_ exposure. **(B)** All DEGs after 5 d- or 10 d-treatment are listed in two columns. **(C)** DEGs induced by C_H_ WS-PM_2.5_ exposure at the 5th day or 10th day were combined, on which GO term/KEGG pathway term functional annotation and enrichment analysis were performed. **(D)** Protein-protein interaction netowrk between proteins of DEGs was predicted by using STRING database.

To clarify the molecular mechanism underling PM_2.5_-induced lifespan and healthspan reduction, RNA-Seq technology was performed on nematodes after a 5 or 10 d-exposure to get the global gene expression profiling. The results showed that a total 35 genes (a combination of DEGs at day 5 and 10) was differently expressed (fold change < 1/2 or >2 together with adjusted *P-*value < 0.05) between WS-PM_2.5_ treated and control nematodes, in which, 31 genes differently expressed at day 5 and 4 genes were differently expressed at day 10 (Figures 2A, B).

GO and KEGG functional annotation as well as the further enrichment analysis helped us to get a profounder insight into the high-level functions of these DEGs, in which 29 out of 35 DEGs were annotated with one or more specific GO or KEGG terms, and 16 functional terms were enriched with statistical significance (*P* < 0.05) (Figure 2C). Almost all the enriched terms are related to fatty acid or lipid metabolism or transport, which indicated that the transport, metabolism and localization of lipids or fatty acids in worms is impacted by WS-PM_2.5_ exposure.

Additionally, PPI network analysis (Figure 2D) indicates that there are at least four clusters of DEGs with distinct interaction network. A cluster including *acs-1, elo-5, elo-6, fat-5, acs-2, sodh-1, mtl-1* directly participate in fatty acid metabolism; a yolk proteins cluster including *vit-1, vit-3, vit-4* are lipids transporters which are homologous to human apolipoproteins. Another cluster including *dpy-14, hch-1, sym-1, C09F9.2, fbn-1, noah-2* are mainly extracellular matrix (ECM) related genes or genes related to the membrane located proteins, most of which participate in cuticle formation, melting cycle, metamorphosis and dauer formation. Some of the rest DEGs such as *ZK6.11, dod-24, pgp-5, sodh-1* participate in innate immune response against exogenous microorganisms.

The GenAge database was searched. As of the last update on September 8, 2022, a total of 889 nematode aging/life-regulation related genes were currently included in the database. The obtained DEGs in our study were compared with the 889 genes, and it was found that only 4 genes related to changes of lifespan (*sams-1, vit-1, elo-5* and *dod-24*) are included in this database, and other genes obtained after 5-d exposure - though 5 genes (*vit-3, vit-4, ZK6.11, sodh-1*, and *mtl-1*) related to lifespan change - are not included in this database; 4 DEGs obtained after 10 d of exposure were not included in the database. Later, RNAi interference technology can be used to further explore its role in nematode lifespan.

#### WS-PM_2.5_ induced shorten lifespan on *daf-2(e1370)* and *daf-16(mu86)*, downgegulated *daf-16* and *hsp-16.2*

**Figure 3:**
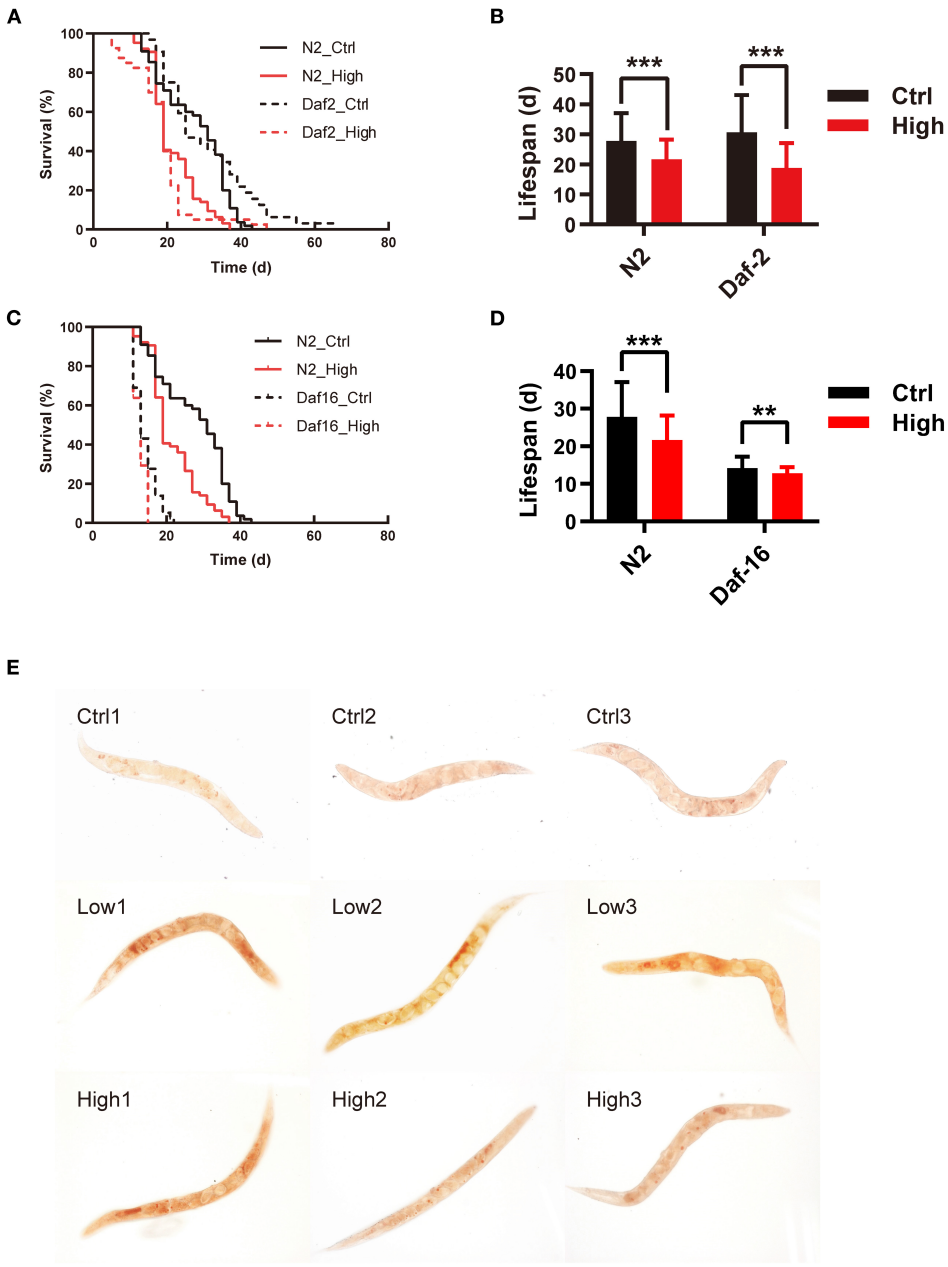
WS-PM_2.5_ exposure affects lifespan of *daf-2*(*e1370*) and *daf-16(mu86)* mutants, and disturbs neutral lipid metabolism in nematodes. **(A–D)** *daf-2(e1370)* **(A, B)**, *daf-16(mu86)* **(C, D)** and N2 nematodes were exposed to WS-PM_2.5_ (with AQI ranging from 100 to 200 and concentration at 119 μg/mL) throughout their life circles. Survival curves **(A, C)** and average lifespan **(B, D)** were recorded and calculated. In all groups, 60 nematodes were randomly selected for experimental manipulation. **(E)** Nematodes were exposed to WS-PM_2.5_ for 5 d. The amount of total neutral lipids of nematodes were indicated by oil red O staining in situ **(E)**. All values are given as mean ± SD. Statistical significance was marked with asterisk on the top of bars of each groups vs. corresponding controls, **p < 0.01, ***p < 0.001. “High” represents C_H_ group.

The IIS pathway plays a key role in the lifespan-related signaling pathway. It has reported that loss of IIS leads to an increased lifespan in nematodes, and IGF-1 promotes aging and shortens lifespan in mammals (30). To reveal the role of IIS in the life-toxic effects of WS-PM_2.5_, *daf-2(e1370)* and *daf-16(mu86)* mutants were compared to N2, and their lifespan were assessed. The results showed that: (i) survival curves of N2 nematodes in WS-PM_2.5_ treatment group could shift to the left, and the average lifespan of nematodes in C_H_ group was shortened by 20.68% (*P* < 0.01) compared with the control group. (ii) WS-PM_2.5_ treatment induced survival curve of *daf-2(e1370)* shifting to the left; compared with the control group, mean lifespan of the nematodes in C_H_ group was shortened by 36.68% (*P* < 0.001) (Figures 3A, B). (iii) After treatment of PM_2.5_, survival curve of *daf-16(mu86)* shifted to the left, and mean lifespan of nematodes in C_H_ group was significantly shortened by 9.18% (*P* < 0.01) compared with the control group (Figures 3C, D). It suggested that WS-PM_2.5_ possess a toxic effect on the lifespan of *C. elegans* through the modulating by insulin signaling pathway. Besides, expression level of *daf-16* was significantly decreased after 5 d-exposure, while *hsp-16.2* showed similar change after 10 d-exposure.

#### Increased reactive oxygen species in nematodes

At low concentrations, ROS play physiological roles in various cellular processes as signaling molecules, such as immune response, oocyte maturation and cuticle formation. When ROS reach a certain threshold, they result in reduced healthspan, shortened lifespan, aging and increased sensitivity to toxicants (31). The nematodes treated with WS-PM_2.5_ were incubated with DCFH-DA so as to investigate the effect on intestinal development with the aid of endpoint of ROS production. The results showed no significant difference between both WS-PM_2.5_ exposure groups (*P* > 0.05), while a significant increase in nematodes of C_H_ group (*P* < 0.05), indicating that PM_2.5_ could induce oxidative damage in the nematodes.

#### Increased lipid content in nematodes induced by WS-PM_2.5_ exposure

According to the RNA-Seq results that WS-PM_2.5_ exposure has an effect on lipid metabolism in nematodes, Oil Red O staining was subsequently used to confirm the lipid distribution in the nematodes. The results showed that after 5 d of WS-PM_2.5_ exposure, the distribution of lipid droplets in the nematodes of the exposed group was more than that of the control group (Figure 3E).

## Discussion

In this study, we concern about the toxic effects of a region-specific PM_2.5_ samples on lifespan in *C. elegans*. To address this question, WS-PM_2.5_ was extracted from PM_2.5_ samples collected in a northern city of China. Our data show that the WS-PM_2.5_ had adverse effects on the development, locomotion behavior, heat tolerance, intestinal fluorescence, lifespan of wild-type (N2) nematodes. Long-term exposure to WS-PM_2.5_ (119 μg/mL) induces significantly shortened lifespan of *daf-2(e1370)* mutant, and decreasing gene expression levels of *daf-16* and *hsp-16.2*. We further used RNA-Sequencing technology to explore the global gene expression changes in *C. elegans* induced by WS-PM_2.5_ exposure and total 35 differentially-expressed genes (DEGs) were screened out. Meanwhile, Gene Ontology (GO) enrichment analysis, KEGG enrichment analysis and homology comparison was conducted and the results show that WS-PM_2.5_ could lead to abnormal lipid metabolism and transportation of nematodes, some of which were homologous to human beings.

Firstly, we found that WS-PM_2.5_ at concentrations around 100 μg/mL did not obviously induce lethal effect to nematodes, but showed a slightly adverse influence on development. Nematodes were sensitive to the changes from environmental factors, reflected on the growth and development of individuals initially, e.g., body length and width. Previous studies have certified the relationship between traffic and coal combustion PM_2.5_ and nematodes poor growth (15, 16), but the concentration is much higher than that we used in this study.

In view of the persistence of PM_2.5_ exposure, lifespan is considered to be an effective index to potentially reflect the long-term effects of toxicants on nematodes (32). We found that average lifespan of nematodes was shortened by about one tenth after treated with 94 and 119 μg/mL WS-PM_2.5_. Given this, some indexes need to be supplemented to evaluate the undesirable effects before death. Locomotion function, food intake capacity of the pharynx pump, lipofuscin deposition as well as stress tolerance (heat stress and oxidative stress) were applied as indicators for healthspan of nematods (25, 33, 34). Under physiological condition, *C. elegans* moves in a sinusoidal fashion by alternately contracting ventral and dorsal muscles, and the movement capacity of *C. elegans* has been repeatedly shown to predict later longevity, in addition nematodes that spend a greater portion of their early life actively pumping or pump at higher rates have a higher chance for longer lifespan (24). WS-PM_2.5_ in our study decelerated the frequencies of head thrash and pharyngeal pump, of which both correlate with poorly quantitative and qualitative changes for the subsequent lifespan and living quality. Beyond that, nematodes possessed a 1/3-shorten lifespan under heat sterss after treated with WS-PM_2.5_ for 5 d or 10 d, indicating that PM_2.5_ could reduce the thermotolerance of *C. elegans*. Heat shock response in *C. elegans* reveals three related neuroendocrine signaling pathways, among which the IIS pathway is the most intensively studied, IIS pathway has been demonstrated to play an important role in the regulation of oxidative stress signal in *C. elegans* (31). IIS pathway is an ancient pathway composed of many genetically conservative genes, including *daf-2, AGE-1/PI3-K, PDK-1, AKT-1/2* and *DAF-16/FoxO* (35). It is the first pathway identified to be closely related to lifespan in *C. elegans*. DAF-2 activation in the intestine itself controls the localization and activation of downstream transcription DAF-16/FOXO, and the intracellular DAF-2-to-DAF-16 signaling in the intestine mediates the major effect on lifespan regulation (35, 36). In our study, 119 μg/mL WS-PM_2.5_ treatment shortened average lifespan by about 1/3 in *daf-2(e1370)*, down-regulated expression levels of *daf-16* and *hsp-16.2* in N2, suggesting that the effect of WS-PM_2.5_ on *C. elegans* lifespan may be mediated by IIS pathway. Small heat shock protein (HSP), e.g., *hsp-16.2*, is regulated in an IIS-dependent manner, and has been proved as a target gene of DAF-16 in *C. elegans*, predicting thermotolerance and the subsequent lifespan changes in *C. elegans* (24, 37–39). In addition, the previous research has been elucidated that DAF-16/FOXO in IIS pathway binds to and transactivates/represses numerous target genes involved in development, lifespan, stress response, dauer formation, metabolism and immunity (35, 36, 40, 41). As the main site for IIS mediated-lifespan regulation, intestine is often found to be a reactive tissue/organ downstream of many lifespan-regulation conditions (35). Excessive intestinal ROS can lead to lipid peroxidation, resulting in irreversible lipofuscin accumulation, and the latter can be considered by proxy as a biomarker of lifespan (24, 42). In line with Sun et al. (16), WS-PM_2.5_ treatment caused ROS as well as lipofuscin accumulation in the intestinal tract. Wang et al. (22) reported that enhanced ROS production induced abnormal high fat accumulation and fatty acid composition, mediated by *fat-5*, which is regulated by *daf-16*. The similar change was found in our study, since almost the functional annoted enriched terms of 35 DEGs are related to lipid/fatty acid metabolism or transport, indicating that WS-PM_2.5_ exposure disturbed lipid/fatty acid transport, metabolism, localization, and subsequently affect lifespan and healthspan. In addition, the abnormal fat storage can be conveniently detected by staining with lipid affinity dyes, such as Oil Red O, of which the results show that neutral lipid droplets deposit in nematodes has been increased significantly by WS-PM_2.5_ exposure (43).

We further performed PPI network analysis and results showed 4 potential DEGs clusters with distinct interaction network. One of the 4 clusters (involving *acs-1, elo-5, elo-6, fat-5, acs-2, sodh-1, mtl-1*) directly participate in fatty acid metabolism; three genes (*vit-1, vit-3, vit-4*) involve in a lipid transporter cluster which are homologoug to human apolipoprotein. Zhang et al. (35) proved that expression levels of *daf-2* and *daf-16* in IIS produce an effect on numerous physiological processes, e.g., intestinal lipid storage increasment, dauer formation promotion during development (hypodermis producing a dauer cuticle). Not alone, another cluster from PPI analysis including *dpy-14, hch-1, sym-1, C09F9.2, fbn-1* and *noah-2* are mainly extracellular matrix (ECM) related genes or genes related to the membrane located proteins, most of which participate in cuticle formation, melting cycle, metamorphosis and dauer formation. Previous study have shown that *daf-2* knock-out may have innate immunity enhancement and bacterial infection resistance (44). We found some of the rest 35 DEGs such as *ZK6.11, dod-24, pgp-5, sodh-1* participate in innate immune response against exogenous microorganisms. Of note, the ultimate outcome of a DAF-16-dependent phenotype depends on its complex regulation network on numerous downstream genes (36). Therefore, the DGEs deserve further validations.

By homology analysis, researchers could estimate the genetic relationship between different species. The more similar the sequences are, the closer they are to each other, and vice versa. In this study, the 35 WS-PM_2.5_-related DEGs acquired by RNA-Sequencing (S1, S2) were queried on WormBase, and a total of 62 transcripts were obtained, all of which were < 50% homologous to 307 human aging/lifespan regulatory genes in WormBase. There may be the following possibilities: (1) 7,663 of 20,000 protein-encoding genes in *C. elegans* genes are homologous to humans (45), of which 164 genes have homology with human aging/lifespan-regulating genes included in GenAge database, excluding the 35 DEGs obtained in this study. That is to say that the 35 genes may be specific genes of nematodes. (2) The 35 DEGs are potentially associated with lifespan related toxic effects caused by WS-PM_2.5_, rather than directly. However, their associations need to be further confirmed, and it is not excluded that they are newly discovered lifespan related genes. (3) It is not ruled out that these 35 DEGs are associated with other non-lifespan related toxic phenotypes (phenotypes not detected in this study) caused by WS-PM_2.5_, that is, genes and their coding products that first showing changes after exposure to WS-PM_2.5_ are not those related with aging/lifespan regulation for human beings. Subsequently, we found that 22 human genes in entire human genome on NCBI were homologous to the 35 nematode DEGs (62 transcription sequences). In addition, we analyzed these 22 homologous genes for disease-related radar analysis, and obtained 20 most relevant diseases. Among them, tumors were found to have the largest number of related genes, and 14 genes were associated with them; metabolic diseases with the highest gene density were 7.28.

Comparing the results of radar analysis with existing epidemiological studies, a study in Nagpur, India showed that the top 5 health risks attributed to PM_2.5_ exposure in the area were acute in children (under 5 years old). Lower respiratory tract infections, ischemic heart disease, chronic obstructive pulmonary disease, stroke, and lung cancer in adults (>25 years old) (46). These 5 diseases are basically included in the disease radar analysis results using homologous genes of human and nematode DEGs (S3, S4). In addition, multiple epidemiological results have reported that PM_2.5_ exposure is related to kidney, blood lipid levels and cardiovascular disease. PM_2.5_ was closely associated with kidney injury, especially chronic kidney disease (CKD), studies showed that PM_2.5_ concentrations were positively associated with the risk of CKD, even harder in younger adults (< 65 years) and men than in elder populations (≥65 years) and women (3). In middle-aged women, long-term exposure to PM_2.5_ can increase the prevalence of cardiac metabolic diseases, and the most relevant is hyperlipoproteinemia; PM_2.5_ exposure is negatively correlated with the protective lipoprotein content in the body and positively correlated with the atherosclerotic lipoprotein content (10, 47, 48). In addition, a meta-analysis showed that there is a close relationship between outdoor PM_2.5_ and gestational diabetes (49).

## Conclusion

From the above results, it suggested that short-term treatment of PM_2.5_ showed toxic effects on normal development of *C. elegans* and long-term treatment showed a negative effect on lifespan, while both short-term and long-term exposure of PM_2.5_ could shorten healthspan involving in IIS pathway-dependent regulation. But beyond that, the regulatory effect may also involve 35 DEGs found in new screening, which may induce disorders in lipid metabolism and transport, and are partially homologous to humans. Disease radar analysis of these 22 homologous genes showed that they were associated with a variety of human diseases, among which tumor genes had the most number of related genes (14/22), and metabolic diseases had the highest intensity (7.28). The results obtained are basically consistent with the results of current epidemiological studies.

## Data availability statement

The datasets presented in this study can be found in online repositories. The names of the repository/repositories and accession number(s) can be found below: https://www.ncbi.nlm.nih.gov/geo/, GSE214215.

## Author contributions

WZ: investigation, data curation, analysis, and writing—original draft. ZL: investigation, data analysis, and editing. GL: conceptualization, resources, and writing—review and editing. LK, HJ, and NZ: investigation. JN and SG: investigation guidance. YZ, XW, and JT: PM_2.5_ extraction. All authors contributed to the article and approved the submitted version.
