# Supplementary material for: PM2.5 induce lifespan reduction, insulin/IGF-1 signaling pathway disruption and lipid metabolism disorder in Caenorhabditis elegans
Source: Front Public Health. 2023 Feb 2;11:1055175. doi: 10.3389/fpubh.2023.1055175 (PMC9932997; doi:10.3389/fpubh.2023.1055175)
Supplement: Supplementary file 1 [file Table_1.DOCX]

**S1. Differentially Expressed Genes and The Functional Annotation at Nematodes after a 5-d PM_2.5_ Exposure**

| Gene ID | David Gene Name | fold change | Q value | Functional Notes | Human Diseases |
| --- | --- | --- | --- | --- | --- |
| WBGENE00006928 | Vitellogenin-4(*vit-4*) | 0.175652985 | 2.3549E-15 | *vit-4* is an ortholog of human CTD-3088G3.8; expected to have lipid transporter activity and nutrient storage activity | No human diseases data for *vit-4* |
| WBGENE00006927 | Vitellogenin-3(*vit-3*) | 0.247963555 | 2.1402E-12 | *vit-3* is an ortholog of human CTD-3088G3.8; is expected to have lipid transporter activity and nutrient activity; expressed in the intestine. | No human diseases data for *vit-3* |
| WBGENE00006925 | Vitellogenin-1(*vit-1*) | 0.275743733 | 4.7932E-28 | *vit-1* is an ortholog of human CTD-3088G3.8; expected to have lipid transporter activity and nutrient storage activity | No human diseases data for *vit-1* |
| WBGENE00008205 | Probable S-adenosylmethionine synthase 1(*sams-1*) | 0.322841959 | 5.9158E-09 | *sams-1* is an ortholog of human MAT1A and MAT2A; it is expected to have ATP-binding activity, metal ion-binding activity and methionine adenosyltransferase activity; participate in the negative regulation of lipid storage; in body wall muscle tissue, Expression in dorsal and ventral nerve cords. | Methionine Adenosyltransferase I/III Deficiency |
| WBGENE00021335 | SaPosin-like Protein family(*spp-23*) | 0.348420158 | 0.002386 | *spp-23* is rich in DA neurons, VA neurons, intestinal and pharyngeal muscle cells; affected by a variety of genes, including daf-16, glp-1, and daf-12; affected by rotenone, tunicamycin, and D-glucose The effects of 13 chemicals included; are expected to encode proteins with the following domains: a sphingolipid B-type domain and a sphingolipid-like domain. | No human diseases data for *spp-23* |
| WBGENE00001244 | Elongation of very long chain fatty acids protein 6(elo-6) | 0.359384477 | 1.2342E-06 | *elo-6* is an ortholog of human ELOVL3 and ELOVL6; it is expected to have transferase activity to transfer acyl groups other than aminoacyl groups; it is expressed in amphid neurons, intestines, nerve rings and vulva. | *No human diseases data for elo-6* |

note：q value (p-adjusted) is the adjusted p value. The smaller the q value, the more significant the difference in gene expression. PM_2.5_ concentration: 119 μg/mL.

**S1. Differentially Expressed Genes and The Functional Annotation at Nematodes after a 5-d PM_2.5_ Exposure**

| Gene ID | David Gene Name | fold change | Q value | Functional Notes | Human Diseases |
| --- | --- | --- | --- | --- | --- |
| WBGENE00001243 | Elongation of very long chain fatty acids protein(elo-5) | 0.406407801 | 0.016789 | *elo-5* is an ortholog of human ELOVL3 and ELOVL6; is expected to have transferase activity, transfer acyl groups other than aminoacyl groups; and is expressed in amphid neurons and intestines. | *No human diseases data for elo-5* |
| WBGENE00022645 | hypothetical protein(ZK6.11) | 0.382412079 | 0.0025727 | ZK6.11 participates in the innate immune response; it is localized on membrane rafts. | *No human diseases data for ZK6.11* |
| WBGENE00018488 | fatty Acid CoA Synthetase family(acs-1) | 0.407705691 | 0.000037883 | *acs-1* is an ortholog of human ACSF2 (acyl-CoA synthetase 2); it is expected to have catalytic activity; participates in the establishment or maintenance of epithelial apical / basal polarity; is expressed in the intestine, nervous system, and somatic gonads | Acyl-CoA Synthetase Fanily, Member2, ACSF2 |
| WBGENE00013073 | H(+) MyoInositol coTransporter(hmit-1.1) | 0.414171446 | 0.00047664 | *hmit-1.1* is an ortholog of human SLC2A13; is expected to have transmembrane transporter activity; it is localized on the apical plasma membrane; and is expressed in intestinal cells. | （Promote glucose transporter） |
| WBGENE00012251 | C-type LECtin(clec-49) | 0.425726854 | 0.0077642 | *clec-49* is an ortholog of human COLEC11, CD302, and LY75; it is expected to have monosaccharide binding activity. | *No human diseases data for clec-49* |
| WBGENE00001397 | Delta(9)-fatty-acid desaturase fat-5(*fat-5*) | 0.469110598 | 0.000011648 | *fat-5* is an ortholog of human SCD (stearoyl-CoA desaturase) and SCD5 (stearoyl-CoA desaturase 5); it is expected to have stearoyl-CoA 9-desaturase activity; participate in Long-chain fatty acid biosynthetic processes and multicellular biological development; expressed in the intestine and tail. | Genes：Stearoyl-CoA desaturase; Stearoyl-CoA desaturase 5 |
| WBGENE00007455 | UDP-glucuronosyltransferase(*ugt-22*) | 0.324974855 | 0.0011031 | *ugt-22* is an ortholog of human UGT3A1 and UGT3A2; it is expected to have glucuronyl transferase activity*.* | No human diseases data for *ugt-22* |

note：q value (p-adjusted) is the adjusted p value. The smaller the q value, the more significant the difference in gene expression. PM_2.5_ concentration: 119 μg/mL.

**S1. Differentially Expressed Genes and The Functional Annotation at Nematodes after a 5-d PM_2.5_ Exposure**

| Gene ID | David Gene Name | fold change | Q value | Functional Notes | Human Diseases |
| --- | --- | --- | --- | --- | --- |
| WBGENE00003482 | Transmembrane cell adhesion receptor mua-3(*mua-3*) | 0.488421969 | 0.040146 | *mua-3* is an ortholog of human FBN1, FBN2, and FBN3; it is expected to have collagen-binding activity and intermediate filament-binding activity; participate in cell-matrix adhesion; localize to hemi desmosome and intermediate filament; express in multiple tissues , Including the epithelial system, muscle cells, rectum and sensory organs; used to study Marfan syndrome; human orthologs of this gene are involved in Weill-Marchesani syndrome, distal joint disease, and eye disease (multiple)*.* |  |
| WBGENE00008476 | hypothetical protein(*E03H4.8*) | 2.027216258 | 0.0010028 | *E03H4.8* is an ortholog of human COPB2 (the coat protein complex subunit β2); it is expected to have structural molecular activity. | Coatomer protein complex, β-2 subunit; COPB 2 |
| WBGENE00009724 | hypothetical protein(*F45D3.4*) | 2.049966026 | 7.4436E-07 | *F45D3.4* is rich in various tissues including body wall muscle tissue, germline precursor cells, subcutaneous tissue, intestine and nervous system; affected by genes including *daf-16, daf-2* and *let-60*; affected by fifteen Effects of various chemicals (including ethanol, 1-methylnicotinamide and rotenone). | No human diseases data for *F45D3.4* |
| WBGENE00003999 | P-GlycoProtein related(*pgp-5*) | 2.067661472 | 0.0042253 | *pgp-5* is an ortholog of human ABCB4, ABCB11 and ABCB1; it is expected to have efflux transmembrane transporter activity; participates in multiple processes: defense response to bacteria, response to drugs and stress response to metal ions; expressed in the intestine. | No human diseases data for *pgp-5* |
| WBGENE00010790 | Alcohol dehydrogenase 1(*sodh-1*) | 2.091299952 | 0.00093967 | *sodh-1* is expected to have alcohol dehydrogenase (NAD) activity and metal ion-binding activity; participate in defense responses against Gram-positive bacteria; and be expressed in subcutaneous tissues, muscle cells, and the nervous system*.* | No human diseases data for *sodh-1* |

note：q value (p-adjusted) is the adjusted p value. The smaller the q value, the more significant the difference in gene expression. PM_2.5_ concentration: 119 μg/mL.

**S1. Differentially Expressed Genes and The Functional Annotation at Nematodes after a 5-d PM_2.5_ Exposure**

| Gene ID | David Gene Name | fold change | Q value | Functional Notes | Human Diseases |
| --- | --- | --- | --- | --- | --- |
| WBGENE00007875 | Downstream Of DAF-16 (regulated by DAF-16) (*dod-24*) | 2.132432389 | 2.0725E-06 | *dod-24* is involved in a defensive response to Gram-negative bacteria. | No human diseases data for *dod-24* |
| WBGENE00009221 | fatty Acid CoA Synthetase family(*acs-2*) | 2.13864941 | 0.0042253 | *acs-2* is an ortholog of human ACSF2; is expected to have catalytic activity; participate in fatty acid metabolism; localize to mitochondria; and is expressed in the digestive system, digestive tract, subcutaneous tissue, muscle system and nervous system. | Gene：Acyl-CoA synthase family, member 2; ACSF 2 |
| WBGENE00001828 | Zinc metalloproteinase nas-34(*hch-1*) | 2.188890826 | 0.00049242 | *hch-1* is expected to have metal endopeptidase activity and zinc ion binding activity; participate in hatching and neuron migration; and be expressed in subcutaneous tissues. | No human diseases data for *hch-1* |
| WBGENE00022816 | FiBrilliN homolog(*fbn-1*) | 2.23225215 | 0.00038773 | *fbn-1* is an ortholog of human FBN3, STAB1 (stabilizing protein 1) and STAB2 (stabilizing protein 2); it is expected to have calcium ion binding activity. | Disease：Marfan Syndrome |
| WBGENE00001075 | DumPY: shorter than wild-type(*dpy-14*) | 2.240001968 | 7.6487E-09 | *dpy-14* is an ortholog of human COL5A1 (collagen Vα1 chain), MARCO and COL11A1 (collagen XIα1 chain); is expected to be a structural component of the stratum corneum; in the epithelial system, head, nervous system and sensory organs expression. | No human diseases data for *dpy-14* |
| WBGENE00009926 | NOmpA Homolog (Drosophila nompA: no mechanoreceptor potential A)(*noah-2*) | 2.286118204 | 0.0035257 | *noah-2* participates in the molting cycle. Loss of noah-2 function through RNAi indicates that NOAH-2 activity is necessary for molting; in addition, NOAH-2 appears to be necessary for embryo and larval development, reproduction, coordinated movement, and overall animal health. | No human diseases data for *noah-2* |
| WBGENE00003473 | Metallothionein-1(*mtl-1*) | 2.312735053 | 1.6126E-06 | *mtl-1* is expected to have cadmium ion binding activity, copper ion binding activity, and zinc ion binding activity; participate in several processes, including response to cadmium ion, response to heat, and response to lead ion; in the intestine and pharynx expression. | No human diseases data for *mtl-1* |

note：q value (p-adjusted) is the adjusted p value. The smaller the q value, the more significant the difference in gene expression. PM_2.5_ concentration: 119 μg/mL.

**S1. Differentially Expressed Genes and The Functional Annotation at Nematodes after a 5-d PM_2.5_ Exposure**

| Gene ID | David Gene Name | fold change | Q value | Functional Notes | Human Diseases |
| --- | --- | --- | --- | --- | --- |
| WBGENE00017498 | Protein Up-regulated in Daf-2(gf)(*pud-4*) | 2.377919704 | 0.00001854 | *pud-4* is expressed in hyp7 syncytia. | No human diseases data for *pud-4* |
| WBGENE00006366 | hypothetical protein (*sym-1*) | 2.301700308 | 1.1428E-07 | *sym-1* is an ortholog of human LRG1; it is localized in extracellular space. Sym-1 encodes a protein containing 15 consecutive leucine-rich repeats (LRRs) that overlap functionally with sym-5 and interact with mec-8. Attachment of extracellular stratum corneum; expression begins at embryonic elongation, and SYM-1 is secreted from the apical subcutaneous surface of the embryo; microarray analysis indicates that sym-1 transcript is reduced in lin-14 loss-of-function mutants, indicating LIN-14 Positively regulates sym-1 expression. | No human diseases data for *sym-1* |
| WBGENE00007479 | hypothetical protein(*C09F9.2*) | 2.429399918 | 0.00015532 | *C09F9.2* is enriched in OLL, PVD and subcutaneous tissue; affected by genes such as daf-2, clk-1 and sir-2.1; affected by 13 chemicals, including methyl hydroxide, 1-methylnicotinamide and rotenone ; Is expected to encode a protein with the following domains: SEA domain, EGF-like domain, trypsin inhibitor-like, cysteine-rich domain, DOMON domain, EGF-like domain, serine protease inhibitor-like superfamily And SEA domain superfamily. | No human diseases data for *C09F9.2* |
| WBGENE00003766 | QWGYGGY-amide(*nlp-28*) | 3.946845375 | 0.011108 | *nlp-28* is enriched in body wall muscle cells, germline, intestinal and pharyngeal muscle cells; affected by genes including daf-16, daf-2 and daf-12; affected by rotenone, manganese chloride and levamisole Effects of 17 chemicals; encodes neuropeptide-like proteins. | No human diseases data for *nlp-28* |
| WBGENE00017484 | hypothetical protein(*F15E6.3*) | 4.36596329 | 0.0078204 | Enriched in the intestine; affected by genes such as daf-16, daf-2, and dpy-10; affected by fifteen chemicals, including 1-methylnicotinamide, methylmercury chloride, and rotenone. | No human diseases data for *F15E6.3* |

note：q value (p-adjusted) is the adjusted p value. The smaller the q value, the more significant the difference in gene expression. PM_2.5_ concentration: 119 μg/mL.

**S1. Differentially Expressed Genes and The Functional Annotation at Nematodes after a 5-d PM_2.5_ Exposure**

| Gene ID | David Gene Name | fold change | Q value | Functional Notes | Human Diseases |
| --- | --- | --- | --- | --- | --- |
| WBGENE00021236 | Protein Up-regulated in Daf-2(gf)(*pud-1.2*) | 2.692226891 | 0.000011648 | *pud-1.2* is expressed in subcutaneous tissue and intestine.Genetic Manual Gene Description PUD-1.2 and PUD-1.1 together encode a protein of unknown function (CE25224); during temperature changes of 15-25 ° C, daf-2 mutants and wild-type adults have PUD-1.2 / PUD-1.1 Protein content is significantly higher; pud-1.2 (RNAi) animals have no obvious mutant phenotype (ie, they have no obvious defects in fertility or germline maintenance); PUD-1.2 / PUD-1.1 proteins are specific for nematodes Family, including paralogs PUD-2.1, PUD-2.2, PUD-3 and PUD-4. | No human diseases data for *pud-1.2* |

note：q value (p-adjusted) is the adjusted p value. The smaller the q value, the more significant the difference in gene expression. PM_2.5_ concentration: 119 μg/mL.
