# Supplementary material for: PM2.5 induce lifespan reduction, insulin/IGF-1 signaling pathway disruption and lipid metabolism disorder in Caenorhabditis elegans
Source: Front Public Health. 2023 Feb 2;11:1055175. doi: 10.3389/fpubh.2023.1055175 (PMC9932997; doi:10.3389/fpubh.2023.1055175)
Supplement: Supplementary file 2 [file Table_2.DOCX]

**S2. Differentially Expressed Genes and The Functional Annotation at Nematodes after a 10-Day PM2.5 Exposure**

| Gene ID | David Gene Name | fold change | Q value | Functional Notes | Human Diseases |
| --- | --- | --- | --- | --- | --- |
| WBGene  00000551 | *clx-1* | 3.435928464 | 1.75E-24 | *clx-1* is affected by genes such as daf-16, daf-2, and glp-1; it is affected by eight chemicals including rotenone, proanthocyanidins, and sodium chloride | No human diseases data for *clx-1* |
| WBGene  00011321 | *fil-1* | 3.177297103 | 0.01363 | *fil-1* shows lipase activity; it is involved in lipid catabolism. | No human diseases data for *fil-1* |
| WBGene  00017892 | *F28B4.3* | 2.236743773 | 2.54E-05 | *F28B4.3* is enriched in intestinal and pharyngeal muscle cells; affected by genes including daf-16, daf-2, and skn-1; 15 chemicals including rotenone, manganese chloride, and mianserin Impact. | No human diseases data for *F28B4.3* |
| WBGene  00044206 | *T26H5.9* | 0.472766593 | 4.34E-07 | *T26H5.9* is rich in digestive system, digestive tract, muscle system and nervous system; affected by genes such as daf-16, daf-2 and glp-1; affected by sixteen chemicals, including methyl hydroxide, 1 -Methylnicotinamide and rotenone. | No human diseases data for *T26H5.9* |

note：q value (p-adjusted) is the adjusted p value. The smaller the q value, the more significant the difference in gene expression. PM2.5 concentration: 119 μg / mL.
