# Supplementary material for: PM2.5 induce lifespan reduction, insulin/IGF-1 signaling pathway disruption and lipid metabolism disorder in Caenorhabditis elegans
Source: Front Public Health. 2023 Feb 2;11:1055175. doi: 10.3389/fpubh.2023.1055175 (PMC9932997; doi:10.3389/fpubh.2023.1055175)
Supplement: Supplementary file 3 [file Table_3.DOCX]

**S3. BLAST results of nematode differential genes and human genes after PM_2.5_ (119 μg / mL) exposure for 5 and 10 d respectively**

| Nematode Gene | Discontigous megablast | | | |
| --- | --- | --- | --- | --- |
|  | Human Homologous Gene | Features | E value | Homology |
| *acs-1.a* | No significant similarity found. |  |  |  |
| *acs-1.b.1* | No significant similarity found. |  |  |  |
| *acs-1.b.2* | No significant similarity found. |  |  |  |
| *acs-1.c* | No significant similarity found. |  |  |  |
| *acs-2* | No significant similarity found. |  |  |  |
| *C09F9.2* | ATP11B | ATPase phospholipid transporter 11B (hypothesis) | 2.6 | 81.40% |
| *clec-49* | No significant similarity found |  |  |  |
| *dod-24.b* | No significant similarity found. |  |  |  |
| *dod-24.a* | No significant similarity found |  |  |  |
| *dpy-14* | PHLPP2 | PH domain and leucine-rich repeat protein phosphatase 2 | 0.042 | 82.00% |
| *E03H4.8* | No significant similarity found |  |  |  |
| *elo-5.a* | ETS1 | ETS proto-oncogene 1, transcription factor | 0.46 | 86.11% |
|  | ELOVL3 | ELOVL fatty acid elongase 3 | 1.6 | 75.00% |
| *elo-5.b* | ETS1 | ETS proto-oncogene 1, transcription factor | 0.47 | 86.11% |
|  | ELOVL3 | ELOVL fatty acid elongase 3 | 1.7 | 75.00% |
| *elo-6* | No significant similarity found in genomic sequence. |  |  |  |

**S3. BLAST results of nematode differential genes and human genes after PM_2.5_ (119 μg / mL) exposure for 5 and 10 d respectively**

| Nematode Gene | Discontigous megablast | | | | |
| --- | --- | --- | --- | --- | --- |
|  | Human Homologous Gene | | Features | E value | Homology |
| *fbn-1.a.1* | No significant similarity found in genomic sequence |  | |  |  |
| *fbn-1.b* | No significant similarity found in genomic sequence |  | |  |  |
| *fbn-1.d* | No significant similarity found in genomic sequence |  | |  |  |
| *fbn-1.e* | No significant similarity found in genomic sequence |  | |  |  |
| *fbn-1.f* | No significant similarity found in genomic sequence |  | |  |  |
| *fbn-1.g* | No significant similarity found in genomic sequence |  | |  |  |
| *fbn-1.h* | No significant similarity found in genomic sequence |  | |  |  |
| *fbn-1.i* | No significant similarity found in genomic sequence |  | |  |  |
| *fbn-1.j* | No significant similarity found in genomic sequence |  | |  |  |
| *fbn-1.k* | No significant similarity found in genomic sequence |  | |  |  |
| *hch-1* | No significant similarity found in genomic sequence |  | |  |  |
| *hmit-1.1* | No significant similarity found |  | |  |  |
| *mtl-1* | No significant similarity found |  | |  |  |
| *mua-3.a* | No significant similarity found |  | |  |  |
| *mua-3.b* | No significant similarity found |  | |  |  |
| *nlp-28* | KRTAP20-1 | Keratin-related protein 20-1 | | 0.15 | 78.43% |
| *noah-2* | No significant similarity found |  | |  |  |

**S3. BLAST results of nematode differential genes and human genes after PM_2.5_ (119 μg / mL) exposure for 5 and 10 d respectively**

| Nematode Gene | Discontigous megablast | | | | | | |
| --- | --- | --- | --- | --- | --- | --- | --- |
|  | Human Homologous Gene | | Features | E value | | Homology | |
| *sams-1.a* | MAT1A | Methionine adenosyl transferase 1A | | | 8e-26 | | 77.16% |
|  | MAT2A | Methionine adenosyl transferase 2A | | | 6e-21 | | 77.54% |
|  | EEA1 | Early endosome antigen 1 | | | 6e-21 | | 66.67% |
| *sams-1.b* | MAT1A | Methionine adenosyl transferase 1A | | | 1e-25 | | 77.16% |
|  | MAT2A | Methionine adenosyl transferase 2A | | | 8e-21 | | 77.54% |
|  | EEA1 | Early endosome antigen 1 | | | 8e-21 | | 66.67% |
| *sams-1.c* | MAT1A | Methionine adenosyl transferase 1A | | | 1e-25 | | 77.16% |
|  | Methionine adenosyl transferase 2A | Methionine adenosyl transferase 2A | | | 8e-21 | | 77.54% |
|  | EEA1 | Early endosome antigen 1 | | | 8e-21 | | 66.67% |
| *ugt-22.a* | No significant similarity found |  | | |  | |  |
| *ugt-22.b* | No significant similarity found |  | | |  | |  |
| *vit-1* | No significant similarity found |  | | |  | |  |
| *vit-3* | No significant similarity found |  | | |  | |  |
| *vit-4* | No significant similarity found |  | | |  | |  |
| *ZK6.11.a.1* | No significant similarity found |  | | |  | |  |
| *ZK6.11.a.2* | STK3 | Serine / threonine kinase 3 | | | 8.5 | | 82.05% |

**S3. BLAST results of nematode differential genes and human genes after PM_2.5_ (119 μg / mL) exposure for 5 and 10 d respectively**

| Nematode Gene | Discontigous megablast | | | | | | |
| --- | --- | --- | --- | --- | --- | --- | --- |
|  | Human Homologous Gene | Features | | | E value | | Homology |
| *ZK6.11.b.1* | No significant similarity found | |  |  | |  | |
| *sym-1* | No significant similarity found | |  |  | |  | |
| *F15E6.3* | No significant similarity found | |  |  | |  | |
| *F45D3.4.a.1* | No significant similarity found | |  |  | |  | |
| *F45D3.4.a.2* | No significant similarity found | |  |  | |  | |
| *F45D3.4.b.1* | GRIPAP1 | | GRIP1-related protein 1 | 1.4 | | 78.00% | |
| *F45D3.4.b.2* | TFE3 | | Binding of transcription factor to IGHM enhancer 3 | 1.4 | | 78.00% | |
| *spp-23* | No significant similarity found | |  |  | |  | |
| *pud-1.2* | No significant similarity found | |  |  | |  | |
| *pud-4.a* | No significant similarity found | |  |  | |  | |
| *pud-4.b* | No significant similarity found | |  |  | |  | |
| *sodh-1* | No significant similarity found | |  |  | |  | |
| *fat-5* | CDRT15L2 | | CMT1A duplicated region transcript 15 like 2 | 7e-06 | | 66.82% | |
|  | SCD | | Stearoyl-CoA desaturase | 6.7 | | 68.00% | |
| *pgp-5.b* | No significant similarity found | |  |  | |  | |

**S3. BLAST results of nematode differential genes and human genes after PM_2.5_ (119 μg / mL) exposure for 5 and 10 d respectively**

| Nematode Gene | Discontigous megablast | | | | | |
| --- | --- | --- | --- | --- | --- | --- |
|  | Human Homologous Gene | Features | E value | | Homology | |
| *pgp-5.a* | GOLGA8G  GOLGA8F  LOC102723534 | golgin A8 family member G  golgin A8 family member F | | 4e-15 | | 67.11% |
|  | ABCB10P4  ABCB10P3 | ATP-binding cassette subfamily B member 10 pseudogene 4  ATP-binding cassette subfamily B member 10 pseudogene 3 | | 4e-15 | | 68.38% |
|  | GOLGA8G | golgin A8 family member G | | 4e-15 | | 68.38% |
|  | ABCB10P4  ABCB10P3 | ATP-binding cassette subfamily B member 10 pseudogene 4  ATP-binding cassette subfamily B member 10 pseudogene 3 | | 4e-15 | | 68.38% |
|  | ABCB4 | ATP-binding box subfamily member B 4 | | 5e-14 | | 76.27% |
|  | ABCB10P1 | ATP-binding cassette subfamily B member 10 pseudogene 1 | | 5e-14 | | 66.78% |
|  | ABCB10P1 | ATP-binding cassette subfamily B member 10 pseudogene 1 | | 5e-14 | | 66.78% |
|  | ABCB11 | ATP-binding box subfamily member B 11 | | 1e-08 | | 73.45% |
|  | ABCB11 | ATP-binding box subfamily member B 11 | | 1e-08 | | 73.45% |
|  | NSUN6 | NOP2/Sun RNA Methyltransferase Family Member 6 | | 2.0 | | 79.17% |

**S3. BLAST results of nematode differential genes and human genes after PM_2.5_ (119 μg / mL) exposure for 5 and 10 d respectively**

| Nmatode Gene | Discontigous megablast | | | |
| --- | --- | --- | --- | --- |
|  | Human Homologous Gene | Features | E value | Homology |
| *clx-1* | No significant similarity found |  |  |  |
| *fil-1* | No significant similarity found |  |  |  |
| *F28B4.3* | No significant similarity found |  |  |  |
| *T26H5.9* | No significant similarity found |  |  |  |
