# Supplementary material for: PM2.5 induce lifespan reduction, insulin/IGF-1 signaling pathway disruption and lipid metabolism disorder in Caenorhabditis elegans
Source: Front Public Health. 2023 Feb 2;11:1055175. doi: 10.3389/fpubh.2023.1055175 (PMC9932997; doi:10.3389/fpubh.2023.1055175)
Supplement: Supplementary file 4 [file Table_4.DOCX]

**S4. Human genetic disease radar analysis table of 35 PM_2.5_-related differential genes related to nematodes**

| Disease | No. of Gene | Name of the Genes | Intensity |
| --- | --- | --- | --- |
| Neoplasms | 14 | ETS1, TFE3, STK3, SCD, PHLPPS2, ABCB4, MAT2A, ABCB11, EEA1, GRIPAP1, MAT1A, ATP11B, GOLGA8G, GOLGA8F | 1.24 |
| Genetic Diseases, Inborn | 10 | ABCB4, ABCB11, ETS1, TFE3, MAT1A, SCD, MAT2A, EEA1, ATP11B, GRIPAP1 | 1.86 |
| Breast Neoplasms | 8 | ETS1, SCD, STK3, PHLPP2, ABCB4, ABCB11, ATP11B | 1.7 |
| Diabetes Mellitus | 8 | SCD, ABCB11, ETS1, ABCB4, TFE3, MAT1A, MAT2A, EEA1 | 2.01 |
| Liver Diseases | 8 | ABCB4, ABCB11, ETS1, MAT1A, SCD, STK3, GRIPAP1, ELOVL3 | 2.63 |
| Metabolic Diseases | 8 | SCD, ABCB4, ABCB11, ETS1, MAT1A, MAT2A, TFE3, EEA1, ABCB4, TFE3, EEA1, ABCB11, PHLPP2,ELOVL3 | 7.28 |
| Diseases | 7 | ETS1, ABCB4, TFE3, EEA1, ABCB11, PHLPP2, ELOVL3 | 2.17 |
| Carcinoma, Hepatocellur | 7 | ETS1, MAT1A, MAT2A, ABCB11, ABCB4, SCD, TFE3 | 2.63 |
| Kidney Diseases | 7 | TFE3, MAT2A, ETS1, SCD, EEA1, ABCB11, PHLPP2 | 2.63 |
| Shock | 7 | ETS1, STK3, MAT2A, ABCB4, SCD, ABCB11, NSUN6 | 3.25 |
| HIV Infections | 7 | EEA1, ETS1, SCD, MAT2A, ABCB4, TFE3, ABCB11 | 1.86 |

Note: Differentially expressed genes after PM_2.5_ (119 μg/mL) exposure for 5 d.

**S4. Human genetic disease radar analysis table of 35 PM_2.5_-related differential genes related to nematodes**

| Disease | No. of Gene | Name of the Genes | Intensity |
| --- | --- | --- | --- |
| Carcinogenesis | 7 | ETS1, SCD, MAT1A, MAT2A, ATK3, TFE3 | 3.41 |
| Motor Neuron Disease | 7 | MAT1A,MAT2A,STK3,EEA1,PHLPP2,GRIPAP1,CDRT15L2 | 1.39 |
| Fibrosis | 6 | ABCB4, ABCB11, ETS1, MAT2A, STK3, EEA1 | 3.56 |
| Growth Disorders | 6 | ETS1, MAT2A, ABCB4, TFE3, EEA1, ABCB11 | 2.32 |
| Heart Failure | 6 | MAT1A, MAT2A, ABCB4, SCD, STK3, ABCB11 | 3.1 |
| Auditory Perceptual Disorders | 6 | MAT1A, MAT2A, SCD, STK3, EEA1, NSUN6 | 2.63 |
| Cardiovascular Diseases | 5 | SCD, MAT1A, ABCB11, MAT2A, ABCB4 | 1.86 |
| Death | 5 | ABCB4, SCD, STK3, ETS1, ABCB11 | 2.01 |
| Dificiency Diseases | 5 | EEA, ETS1, MAT1A, STK3, ABCB11 | 2.17 |

Note: Differentially expressed genes after PM_2.5_ (119 μg/mL) exposure for 5 d.
