# Supplementary material for: PM2.5 induce lifespan reduction, insulin/IGF-1 signaling pathway disruption and lipid metabolism disorder in Caenorhabditis elegans
Source: Front Public Health. 2023 Feb 2;11:1055175. doi: 10.3389/fpubh.2023.1055175 (PMC9932997; doi:10.3389/fpubh.2023.1055175)
Supplement: Supplementary file 6 [file Presentation_1.PPTX]

## Slide 1
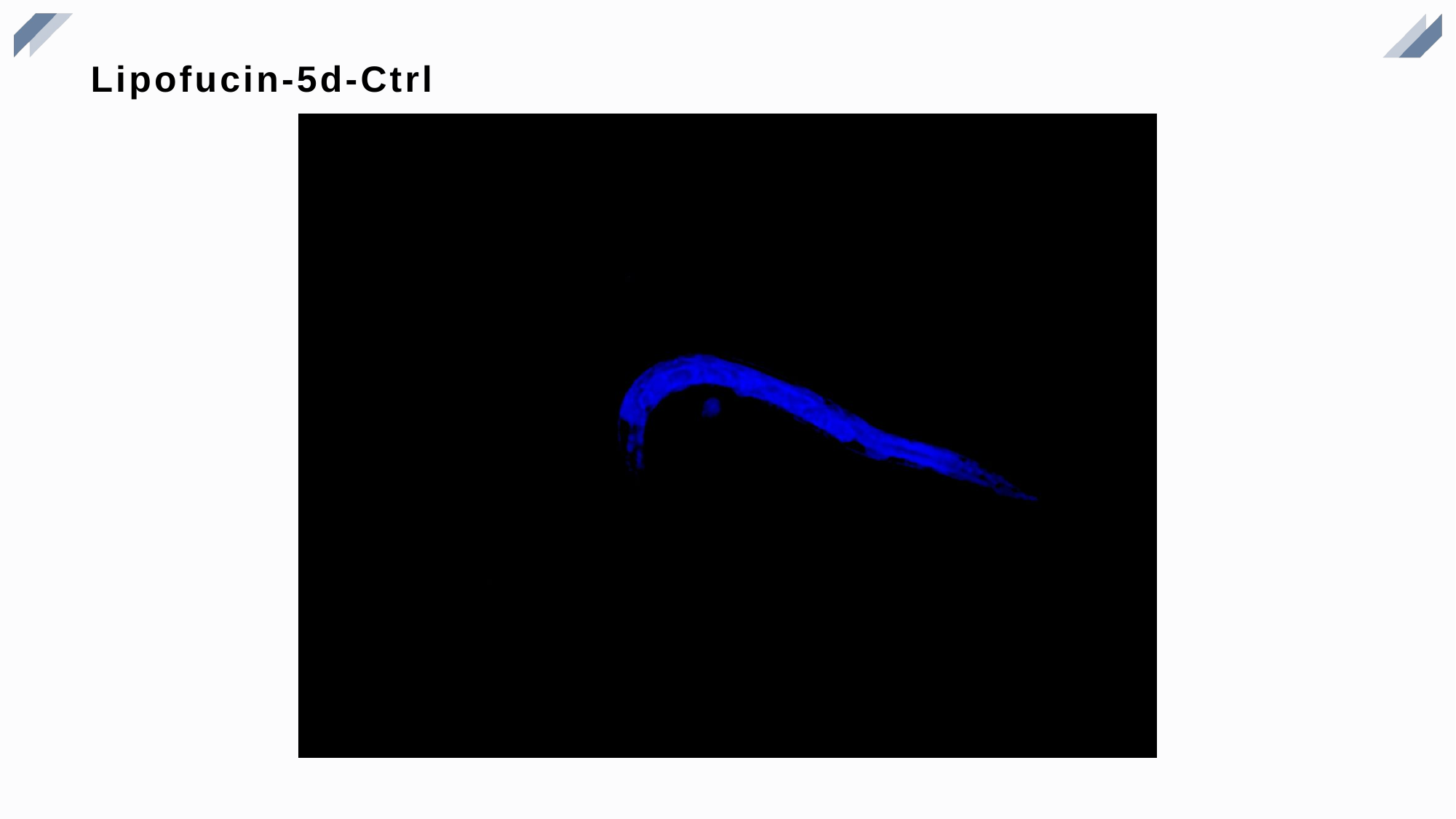

# Lipofucin-5d-Ctrl

## Slide 2
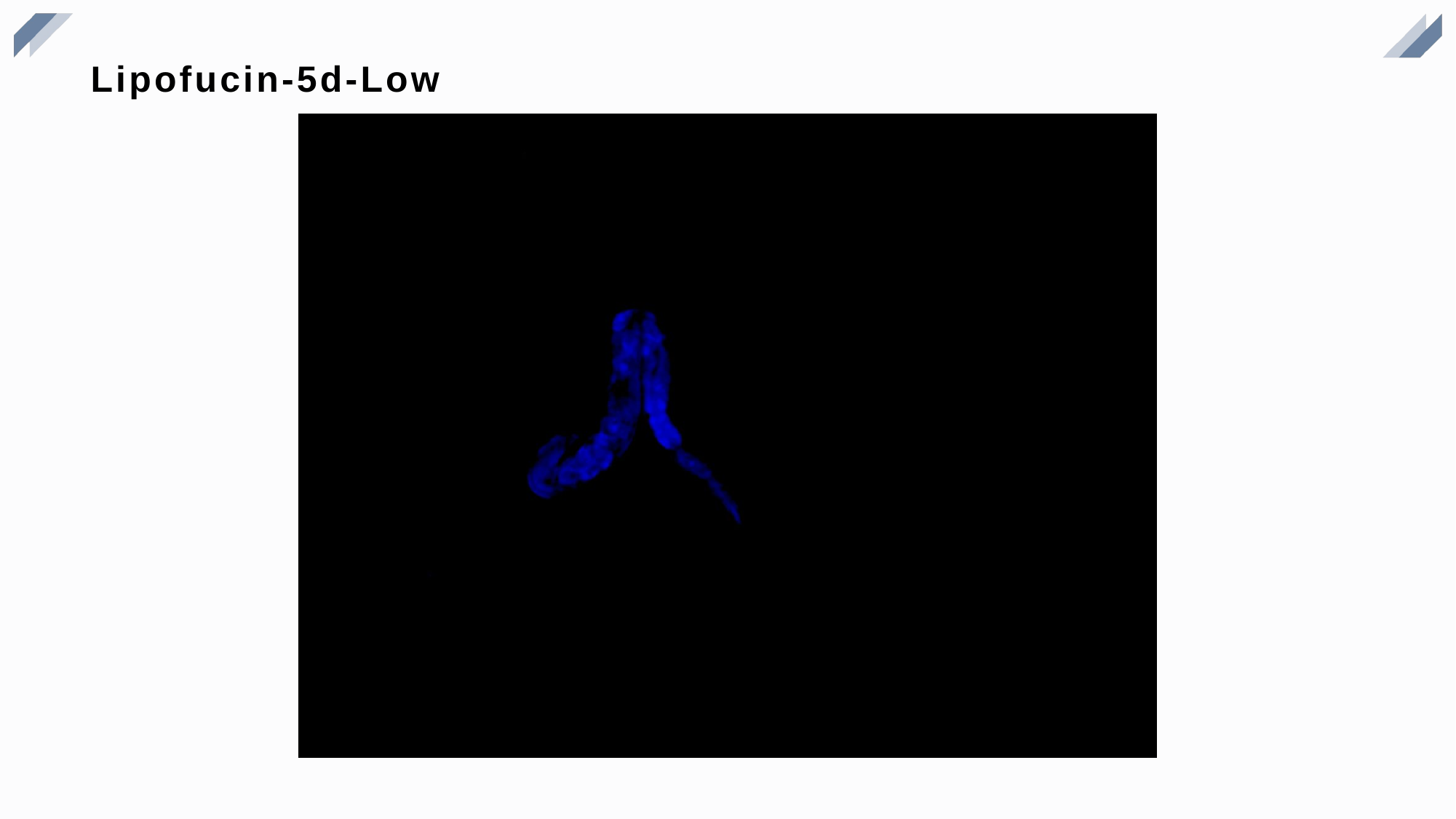

# Lipofucin-5d-Low

## Slide 3
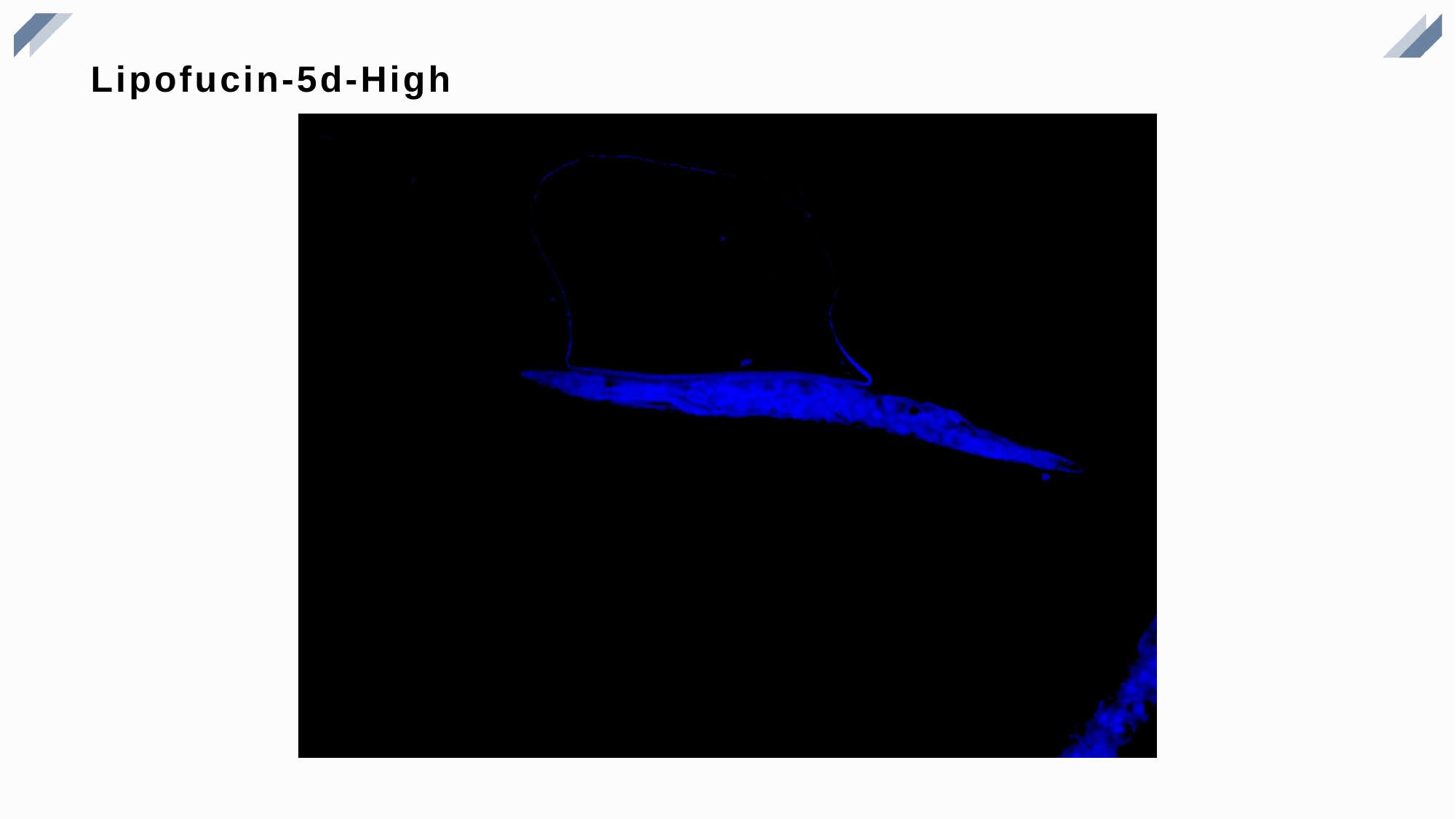

# Lipofucin-5d-High

## Slide 4
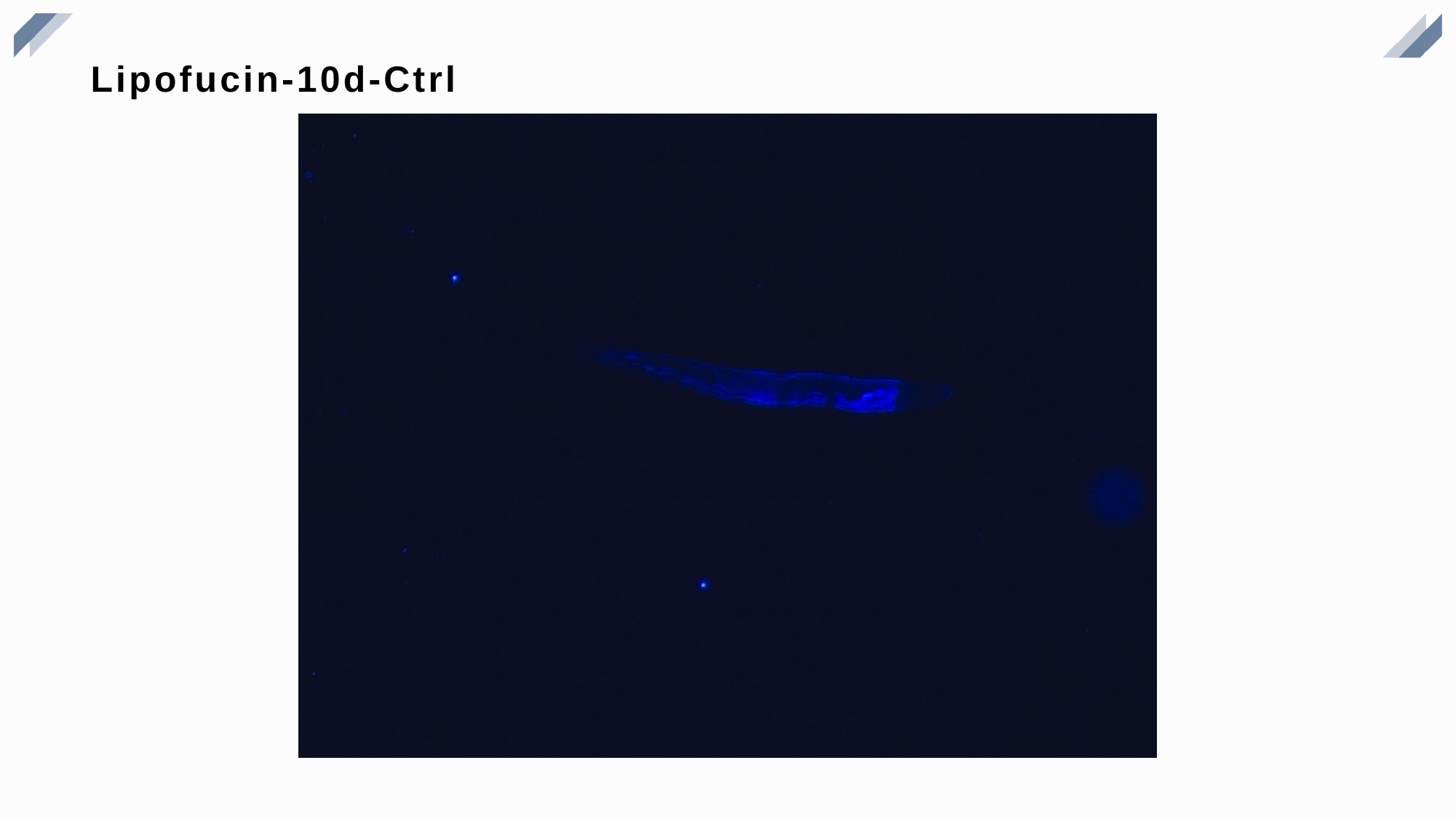

# Lipofucin-10d-Ctrl

## Slide 5
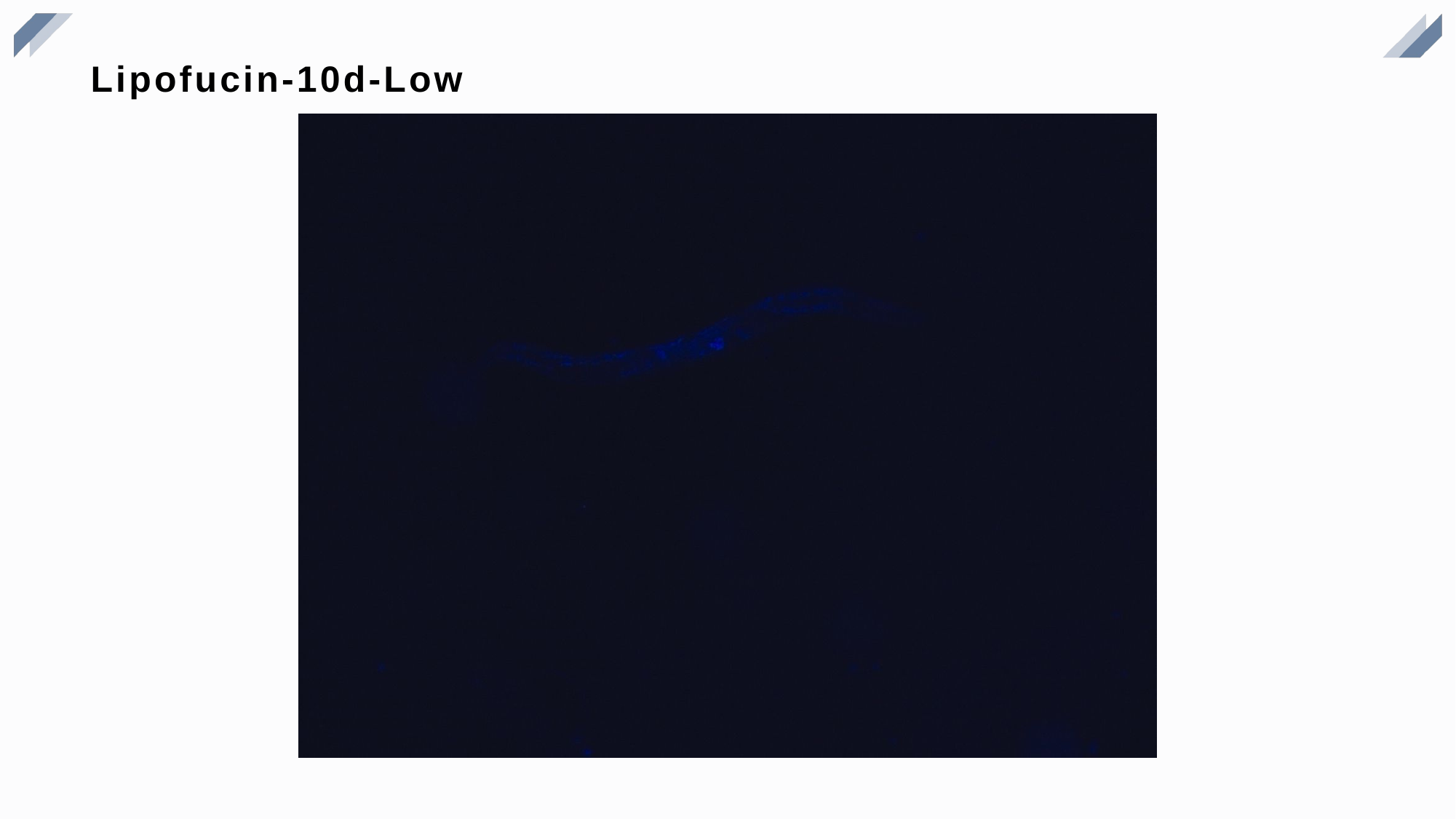

# Lipofucin-10d-Low

## Slide 6
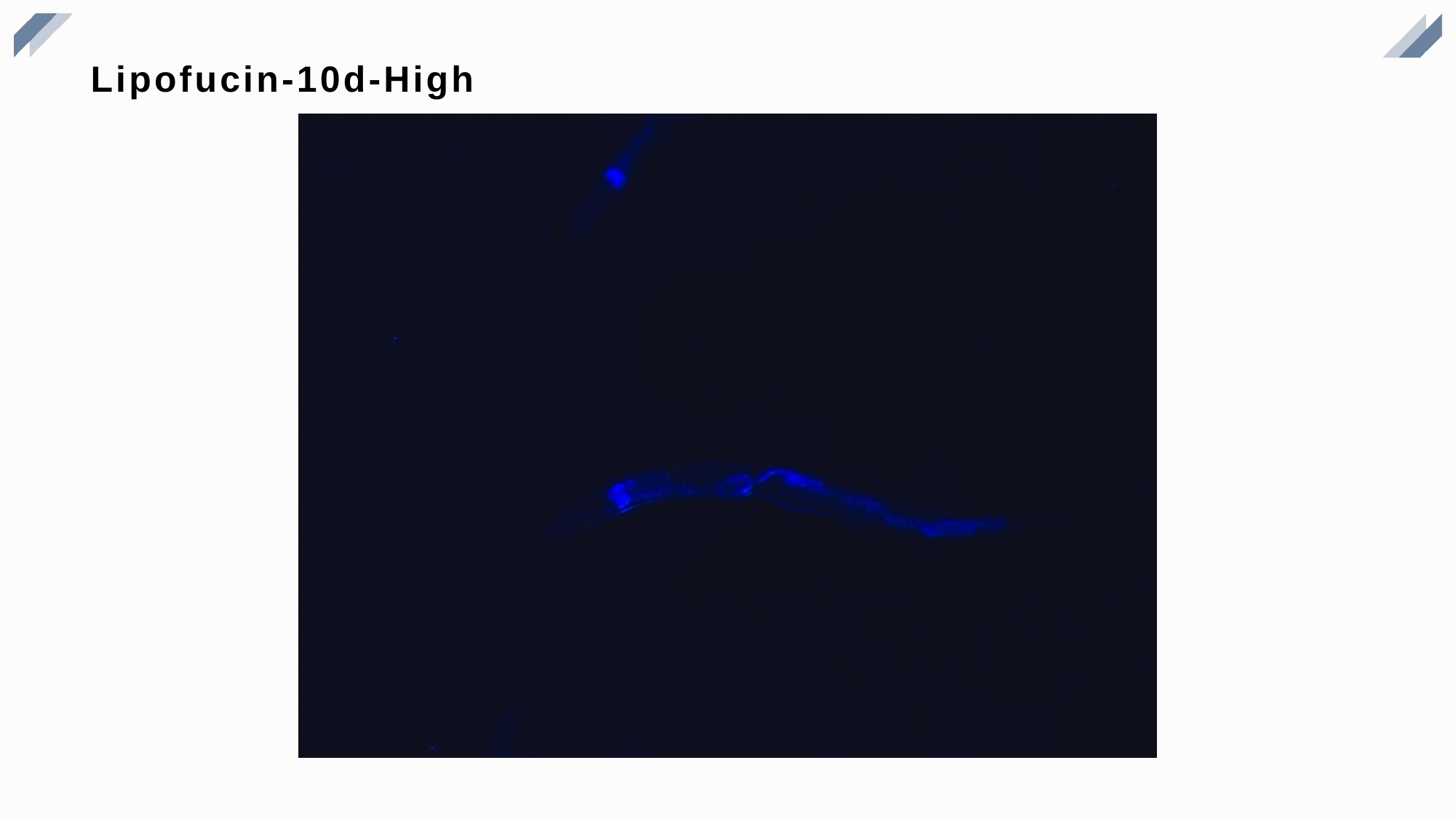

# Lipofucin-10d-High

## Slide 7
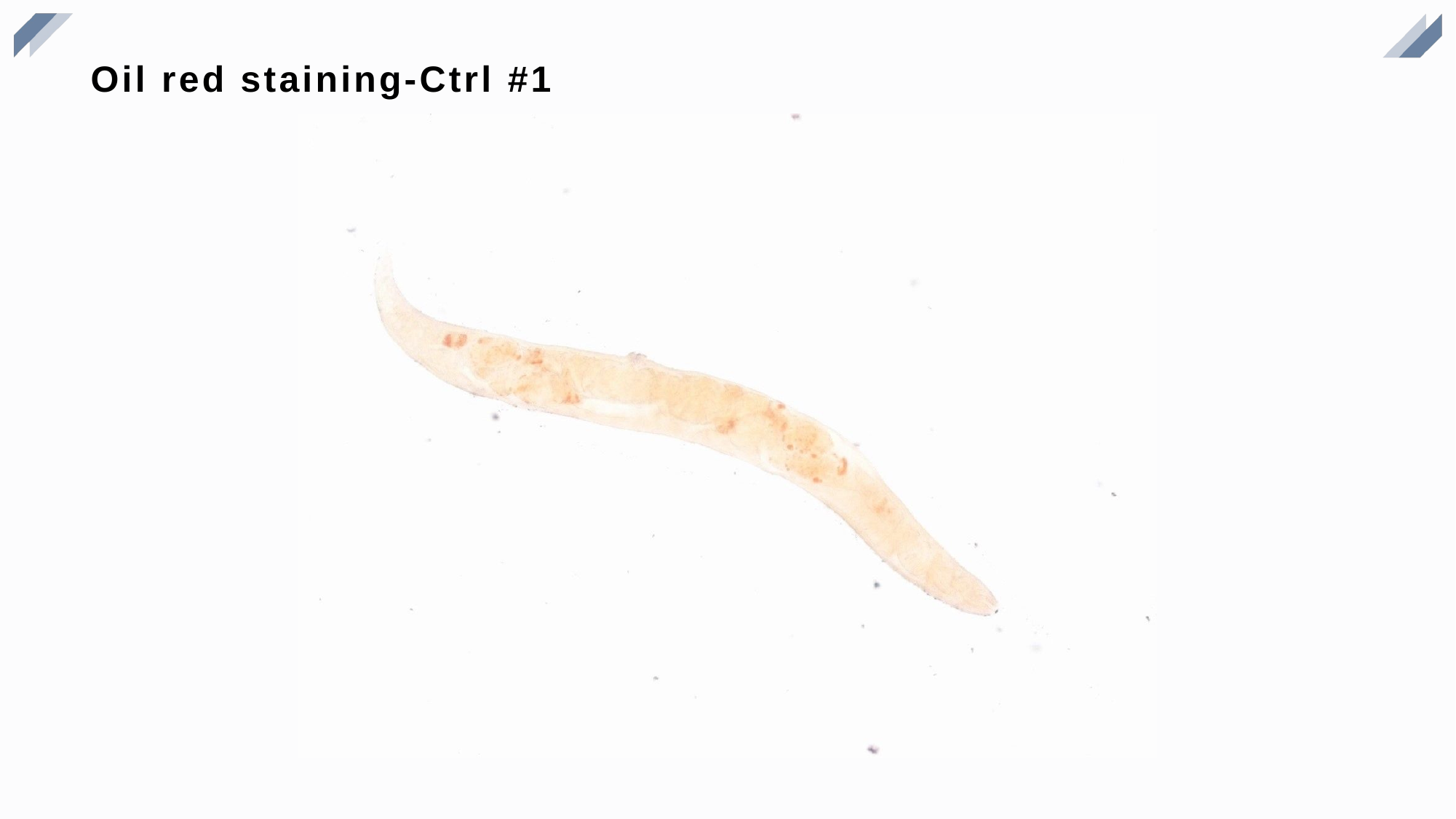

# Oil red staining-Ctrl #1

## Slide 8
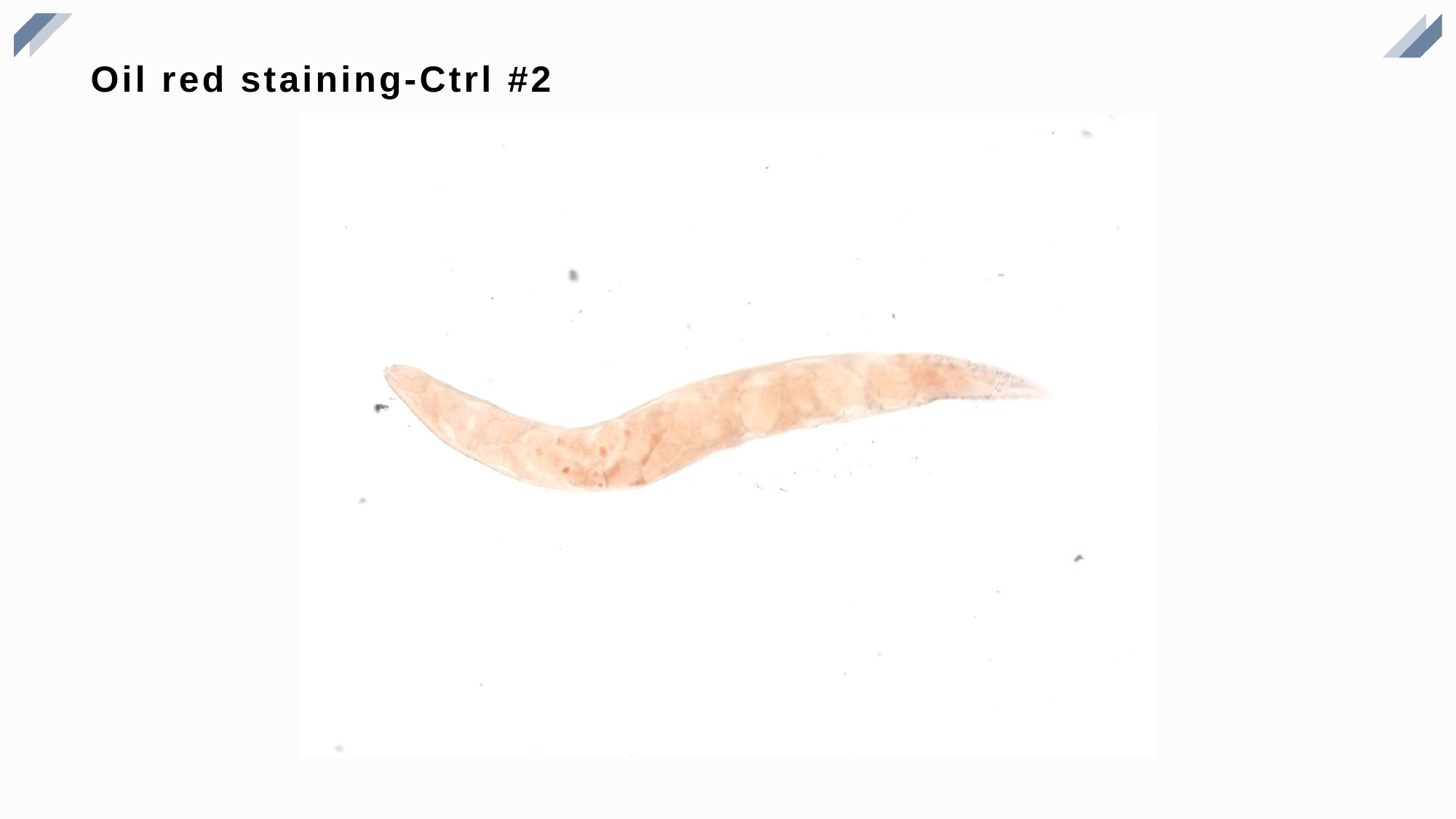

# Oil red staining-Ctrl #2

## Slide 9
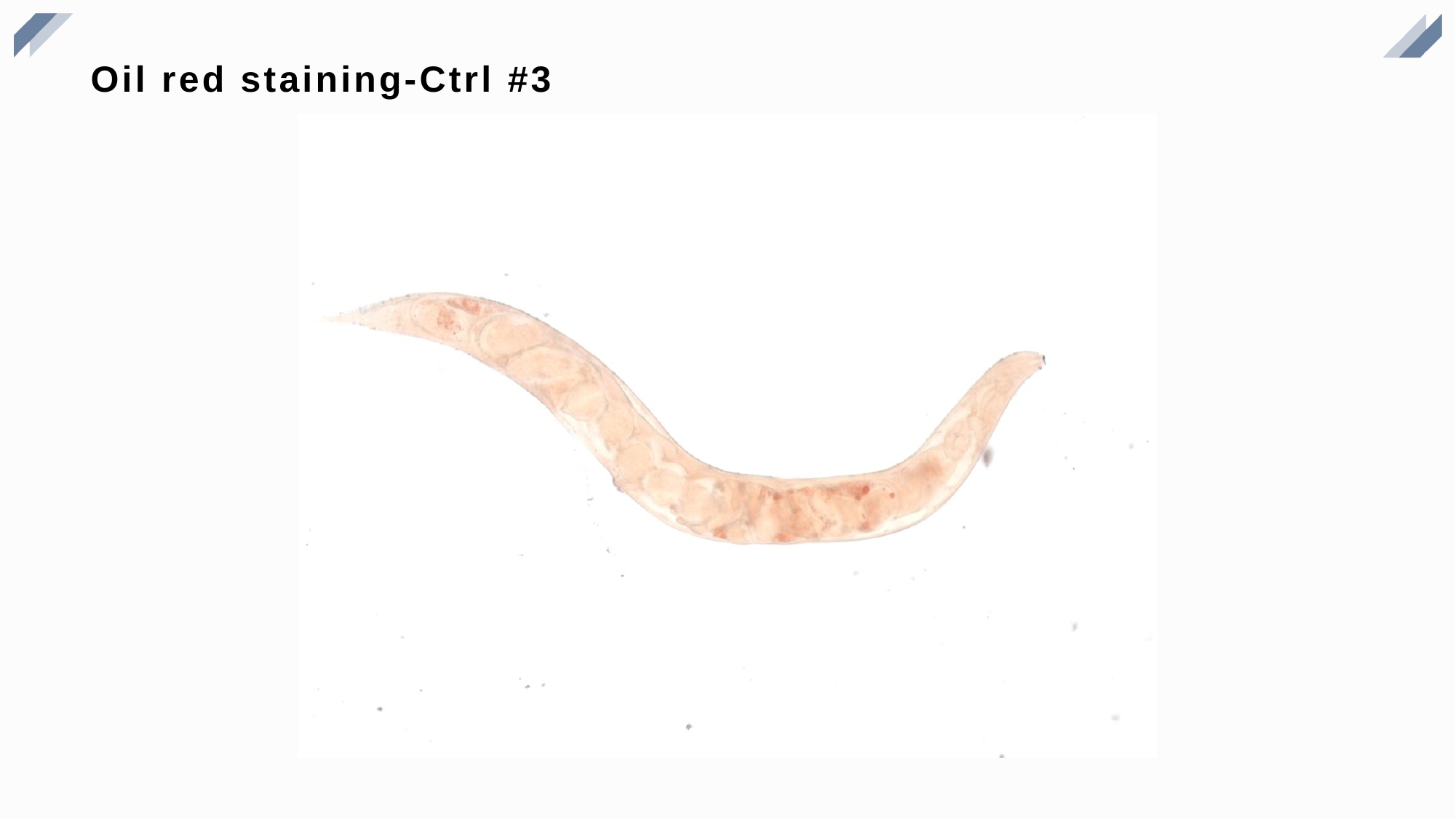

# Oil red staining-Ctrl #3

## Slide 10
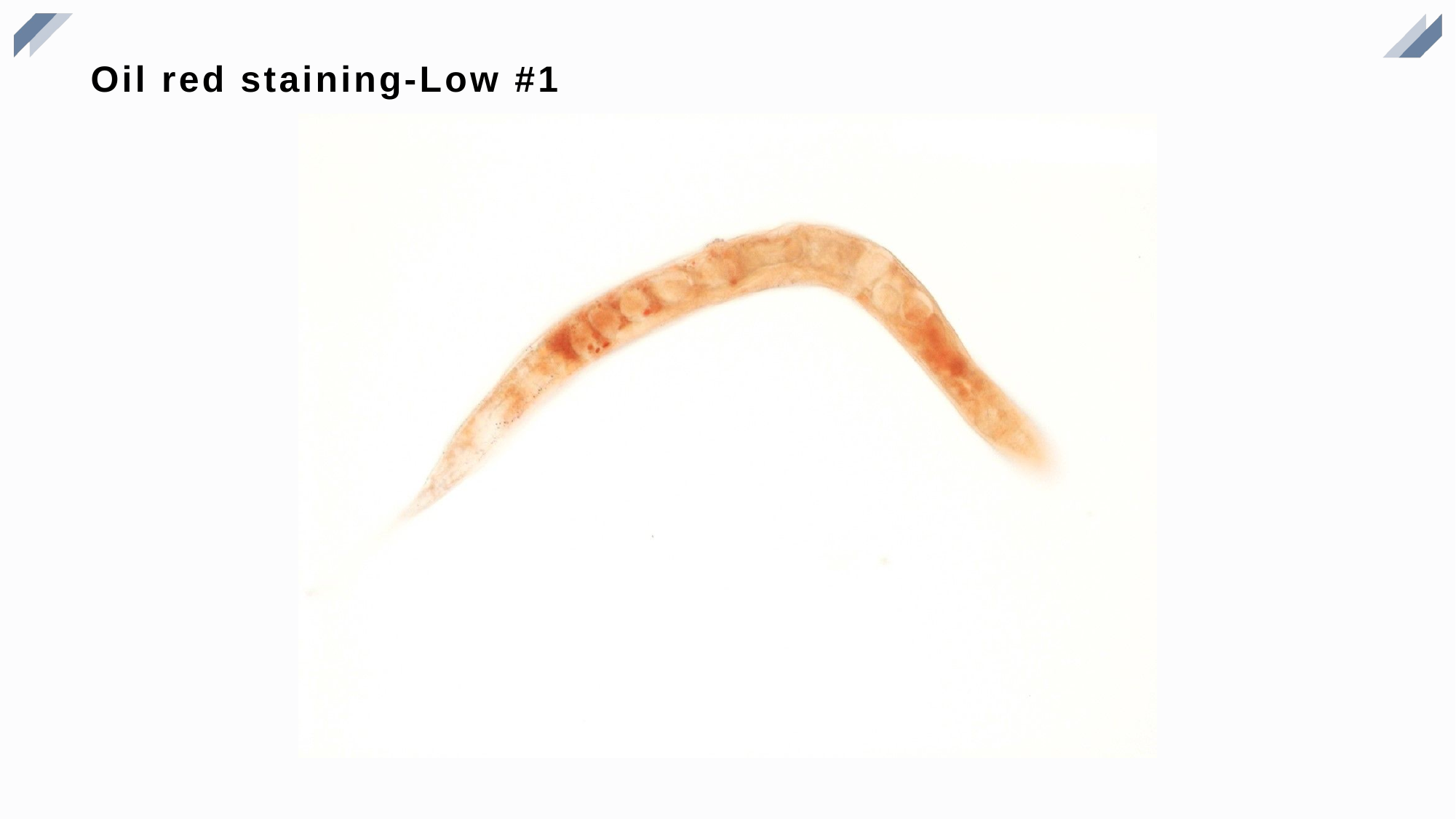

# Oil red staining-Low #1

## Slide 11
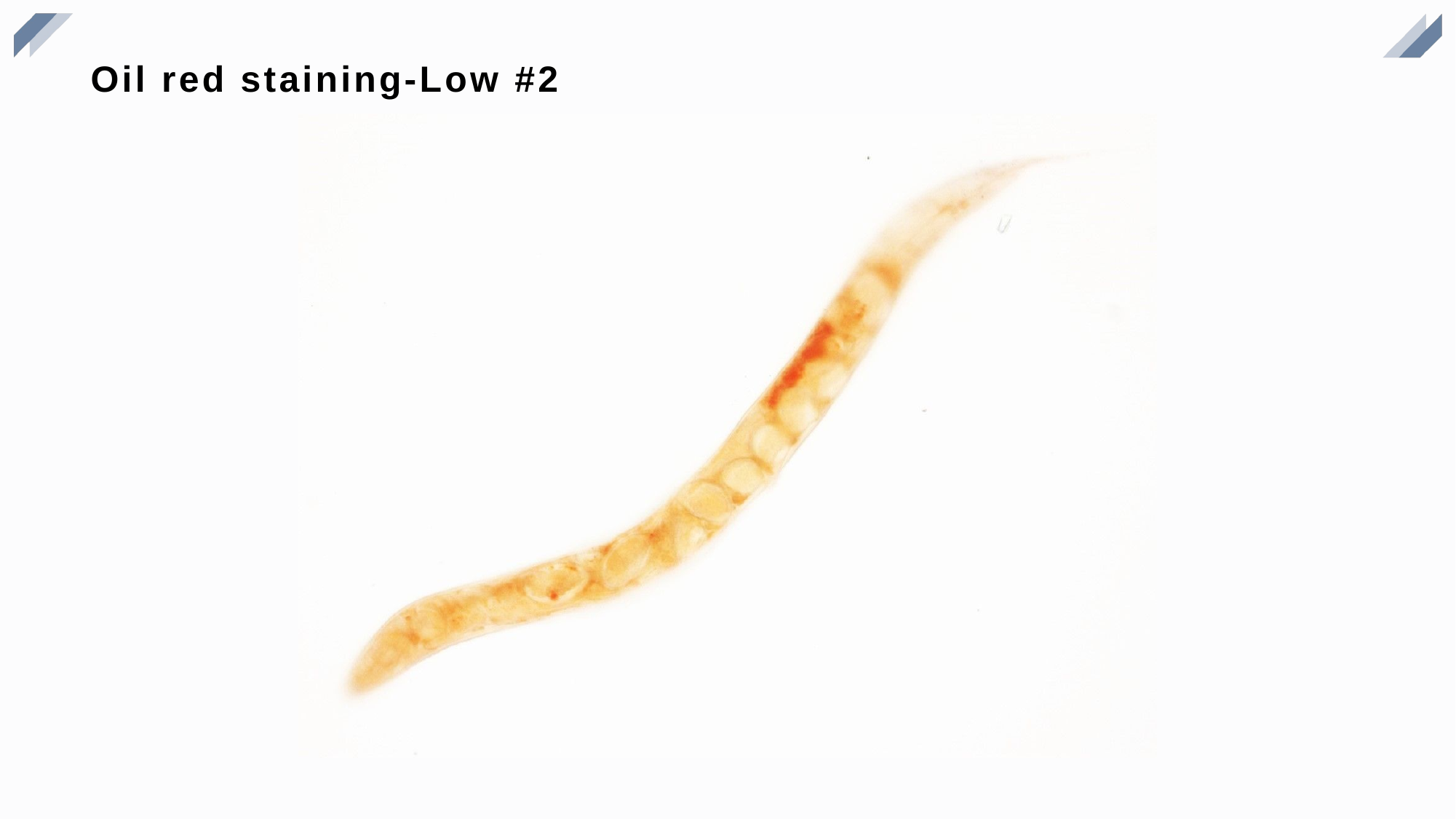

# Oil red staining-Low #2

## Slide 12
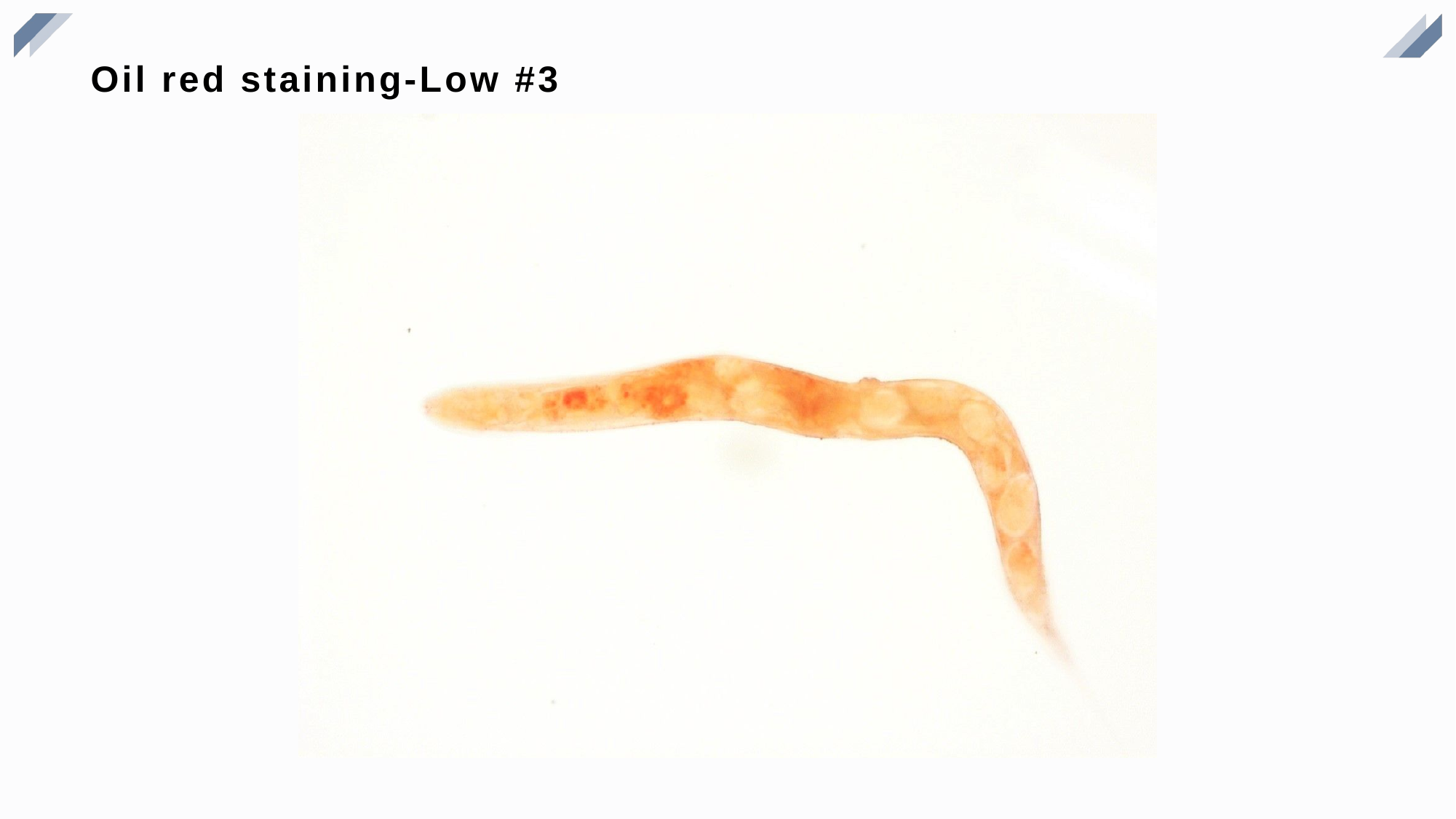

# Oil red staining-Low #3

## Slide 13
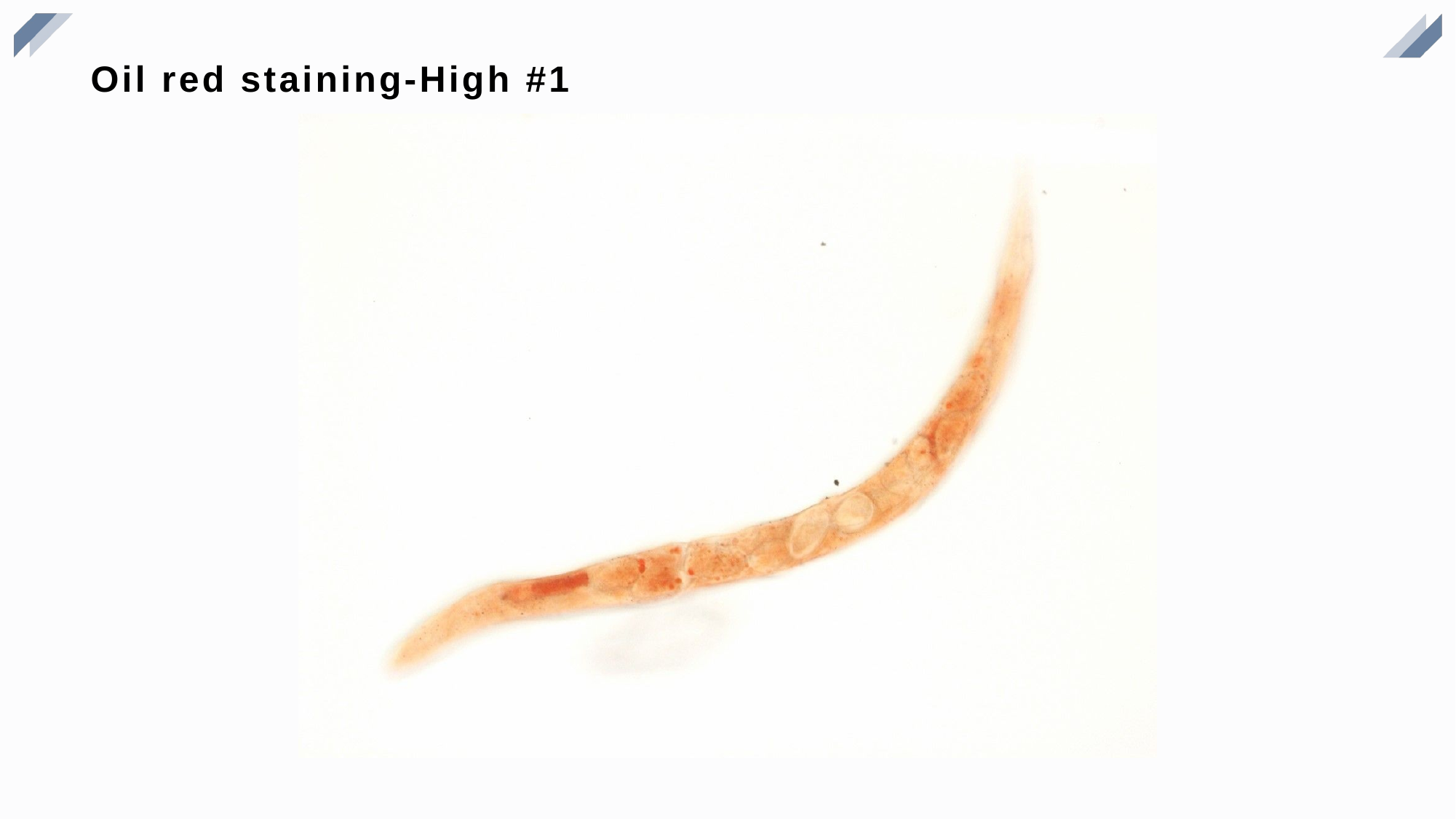

# Oil red staining-High #1

## Slide 14
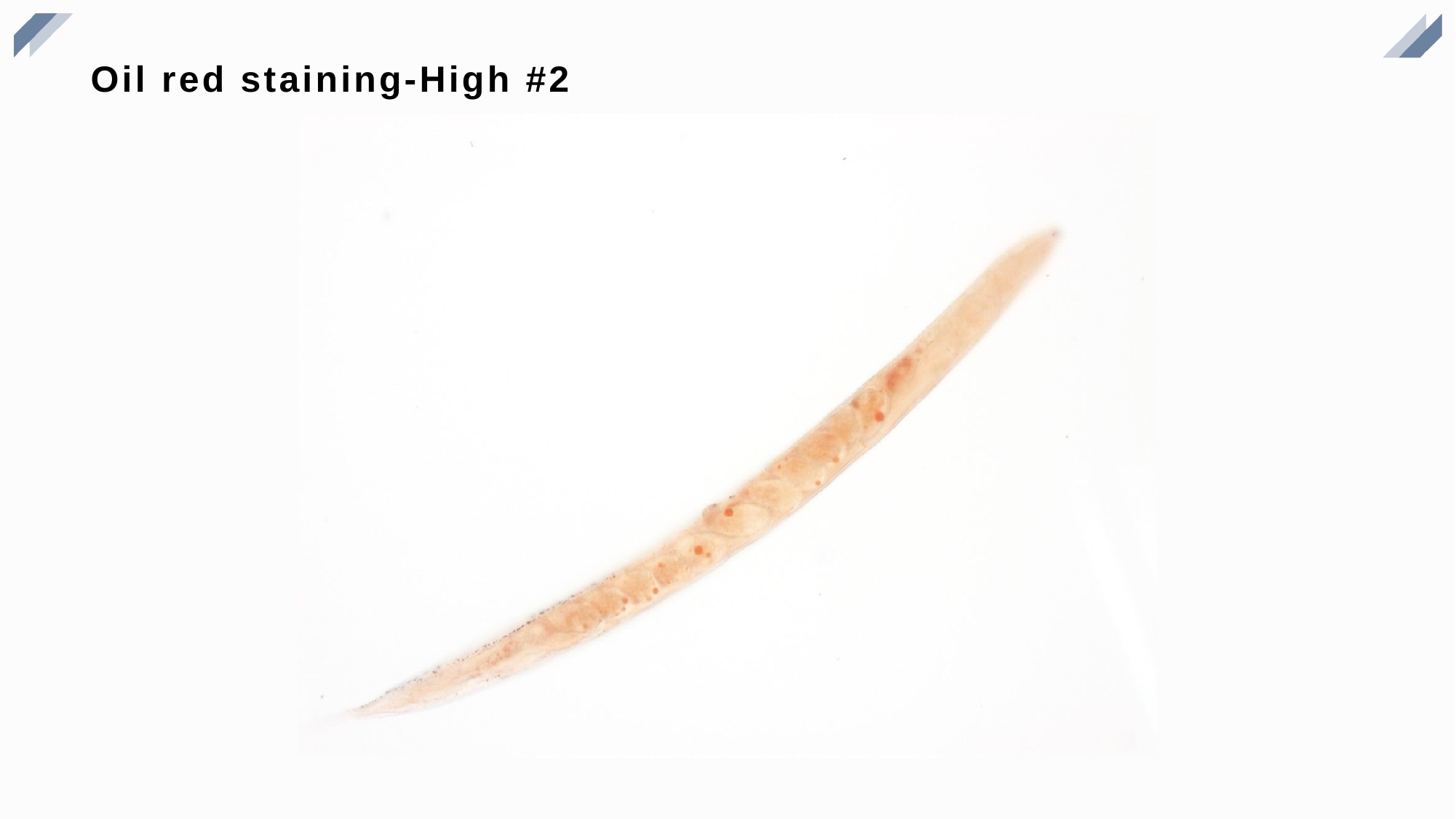

# Oil red staining-High #2

## Slide 15
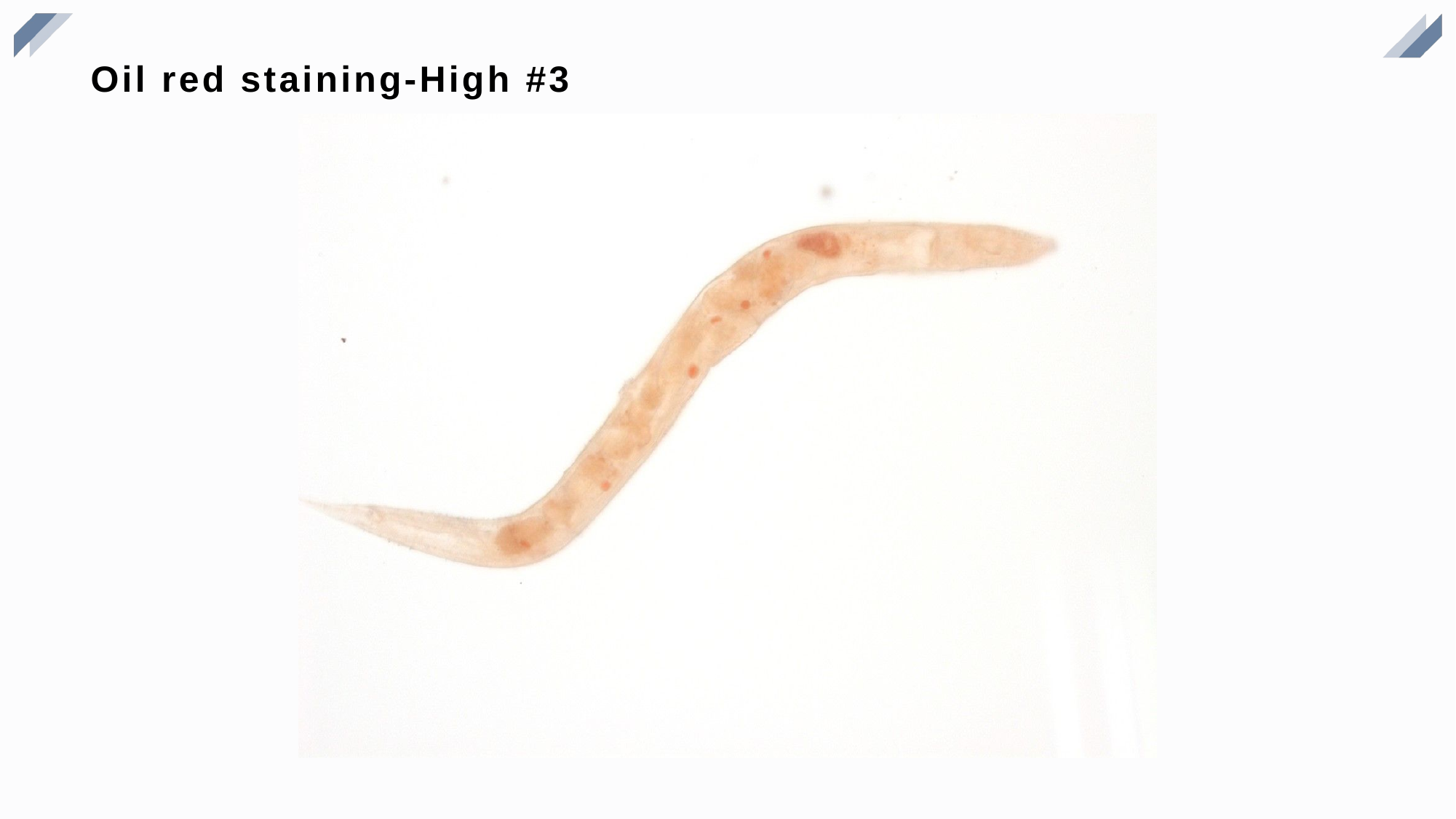

# Oil red staining-High #3
